# Comparative Transcriptome Analysis of Onion in Response to Infection by *Alternaria porri* (Ellis) Cifferi

**DOI:** 10.3389/fpls.2022.857306

**Published:** 2022-04-11

**Authors:** Kiran Khandagale, Praveen Roylawar, Onkar Kulkarni, Pravin Khambalkar, Avinash Ade, Abhijeet Kulkarni, Major Singh, Suresh Gawande

**Affiliations:** ^1^Department of Botany, Savitribai Phule Pune University, Pune, India; ^2^Department of Botany, Sangamner Nagarpalika Arts, D. J. Malpani Commerce, B. N. Sarda Science College, Sangamner, India; ^3^Bioinformatics Centre, Savitribai Phule Pune University, Pune, India; ^4^ICAR-Directorate of Onion and Garlic Research (DOGR), Pune, India

**Keywords:** onion, purple blotch, *RNAseq*, *Alternaria porri*, antioxidant, PR proteins

## Abstract

Purple blotch (PB) is one of the most destructive foliar diseases of onion and other alliums, caused by a necrotrophic fungal pathogen *Alternaria porri*. There are no reports on the molecular response of onion to PB infection. To elucidate the response of onion to *A. porri* infection, we consequently carried out an *RNAseq* analysis of the resistant (Arka Kalyan; AK) and susceptible (Agrifound rose; AFR) genotype after an artificial infection. Through differential expression analyses between control and pathogen-treated plants, we identified 8,064 upregulated and 248 downregulated genes in AFR, while 832 upregulated and 564 downregulated genes were identified in AK. A further significant reprogramming in the gene expression profile was also demonstrated by a functional annotation analysis. Gene ontology (GO) terms, which are particularly involved in defense responses and signaling, are overrepresented in current analyses such as “oxidoreductase activity,” “chitin catabolic processes,” and “defense response.” Several key plant defense genes were differentially expressed on *A. porri* infection, which includes pathogenesis-related (PR) proteins, receptor-like kinases, phytohormone signaling, cell-wall integrity, cytochrome P450 monooxygenases, and transcription factors. Some of the genes were exclusively overexpressed in resistant genotype, namely, *GABA transporter1*, *ankyrin repeat domain-containing protein*, *xyloglucan endotransglucosylase/hydrolase*, and *PR-5* (*thaumatin-like*). Antioxidant enzyme activities were observed to be increased after infection in both genotypes but higher activity was found in the resistant genotype, AK. This is the first report of transcriptome profiling in onion in response to PB infection and will serve as a resource for future studies to elucidate the molecular mechanism of onion-*A. porri* interaction and to improve PB resistance in onions.

## Introduction

Purple blotch (PB) is one of the most destructive foliar diseases in onion, caused by *Alternaria porri* (Ellis) Cifferi. Purple blotch is prevalent in all onion-growing countries in the world (Kareem et al., 2012). Symptoms of the disease encompass small chlorotic, water-soaked brown lesions on leaves, and as infection advances lesion enlarges with purple spots, in a humid climate, these lesions are occasionally seen covered with black-purple spores. In India, its high degree of severity is evidenced by heavy yield losses in both bulb and seed crops, which vary between 2.5 and 85% (Tripathy et al., 2013; Veeraghanti et al., 2017). For the management of PB, mainly chemical fungicides were used, but its excessive and repeated use led to an increase in production cost, deterioration of the environment, and development of resistance in pathogen. Although several bioagents have been reported to antagonize *A. porri* (Tyagi et al., 1990; Prakasam and Sharma, 2012; Abdel-Hafez et al., 2014; Gothandapani et al., 2015), their commercial utility at the field level is limited. Therefore, in such condition, improvement of host resistance by the selection, breeding, and biotechnological tools will be the best sustainable approach (Dar et al., 2020).

To date, limited sources of PB resistance have been reported in onions (Ganesh and Veeregowda, 2007; Behera et al., 2013; Tripathy et al., 2013; Nanda et al., 2016). The PB resistance in onions is controlled by additive and non-additive gene effects (Evoor et al., 2007) and a single dominant qualitative gene (Abubakar and Ado, 2008). Furthermore, the novel PB-resistant gene *ApR1* was mapped using markers (AcSSR7 and ApR-450) linked to PB resistance in the F_2_ population developed from Arka Kalyan (AK) and Agrifound rose (AFR) (Chand et al., 2018). Barring these few studies, the mechanism of PB resistance at a molecular level is not fully known.

Plants respond to the invasion of the pathogen through transcriptional regulation, and large-scale approaches such as transcriptional analysis and genome-wide association studies have been widely applied to uncover the molecular mechanism of plant defense mechanisms (Gupta et al., 2016; Bartoli and Roux, 2017; Zhu et al., 2017; Juliana et al., 2018). Significant advances in understanding defense processes have been made in many crops, and the RNAseq for transcriptome analysis has become a powerful tool to investigate plant disease responses in plants where a high-quality genome sequence is not available (Ghodke et al., 2020; Khandagale et al., 2020; Kim et al., 2021).

Onion (*Allium cepa* L.), from the Amaryllidaceae family, is a vegetable of paramount importance in India and other parts of the world (Chinnappareddy et al., 2013). India is the second largest producer of onion among the major onion producer countries of the world with a production of 26.7 million tons from the 1.4-million-hectare area with a yield of 18.6 ton/ha in 2020.^1^ Although PB is inflicting significant losses to this economically important crop, very less research is still being performed into the molecular response against in a known PB-resistant cultivar. Therefore, in this study, we examined biochemical and molecular responses in resistant AK and susceptible AFR genotypes when infected by *A. porri.*

## Materials and Methods

### Plant Material and Pathogen

For this study, PB-resistant (AK) and susceptible (AFR) varieties were selected for this study (Chand et al., 2018). Seeds of AK and AFR were procured from the Indian Institute of Horticulture Research, Bangalore and National Horticultural Research and Development Foundation, New Delhi, respectively. Pure culture of *A. porri* was isolated from the experimental field of ICAR-DOGR and is maintained on potato dextrose agar (PDA). The identification of the pathogen was ensured by amplifying the ITS region of the fungal DNA with the primers ITS1 5’TCCGTAGGTGAACCTGCGG3’ and ITS4 5’CTGTTGGTTTCTTTTCCTCCGC3’ according to White et al. (1990). The amplified fragment was purified and sequenced, and the resulting DNA sequence was aligned with GenBank using BLASTN at the National Center for Biotechnology Information (NCBI) database which showed 100% identity with *A. porri* (LC440611.1) and submitted in NCBI GenBank (OM131604).

### Experiment

Seeds of both genotypes were surface sterilized with sodium hypochlorite (4%) for 10 min and 70% alcohol for 30 s followed by a three-time wash with distilled water. The seeds were sown in plastic pots with sterilized soil, and after 45 days, the seedlings were transplanted into fresh pots with similar sterilized soil. Each pot contained 4 plants, 10 pots were used for each replication, and the experiment was performed in triplicate. To prepare a fungal pathogen spore suspension, mycelial plugs from *A. porri* culture plate were transferred to a fresh PDA and incubated at 25 ± 2°C for 10 days. The fungal culture was scraped and mixed with distilled water to obtain a final spore concentration of 10^6^ spores/ml. After 30 days of transplantation, the plants were inoculated with a pathogen spore suspension. The leaves were sprayed with pathogen spore suspension and allowed to dry for 2 h, and then the pots were covered with a transparent polythene bag for 24 h to maintain humidity. Control plants were treated similarly, except for pathogen spore suspension; they were sprayed with sterile water. The pots were kept in the greenhouse at 25 ± 2°C, and the leaf tissue was harvested from control and inoculated plants at 5 dpi (day postinoculation), frozen in liquid nitrogen, and stored in –80°C until further studies.

### RNA Isolation and Library Preparation and RNA Sequencing

The total RNA of each control (AKC, AFRC) and infected (AKT, AFRT) plant was extracted using the RNeasy Plant Mini Kit (Qiagen). A pool of three plants was used for RNA isolation, and as one replicate, such two replicates were used in this study. Total RNA of each replicate was quantified using NanoDrop 1000 (Thermo Fisher Scientific, Waltham, MA, United States), and the integrity was assessed on a 1% agarose gel. Further integrity of the isolated RNA was assessed by Agilent 2100 Bioanalyzer (Agilent Technologies, Palo Alto, CA, United States). An equal amount of high-quality RNA having a RIN value above 7 from three plants of each replicate was pooled and used for library preparation. A total of 8 (AKC1, AKC2, AKT1, AKT2, AFRC1, AFRC2, AFRT1, and AFRT2) next-generation sequencing libraries were constructed according to the manufacturer’s protocol (NEBNext^®^ UltraTM RNA Library Prep Kit for Illumina^®^). These libraries were sequenced in both directions with a read length of 150 × 2 using the Illumina HiSeq 2500 platform.

### *De novo* Assembly and Sequence Annotation

The quality check for high throughput sequencing reads was performed using FASTQC Toolkit version 0.11.9.^2^ The low-quality reads (*Q*-value ≤ 20) were discarded using cutadapt version 2.8,^3^ and the remaining reads were considered clean reads. The obtained clean reads of the AK and AFR varieties were assembled using Trinity assembler version 2.12.0^4^ to build a mega-assembly. The transcripts were then clustered using the CD-HIT version 4.8.1^5^ tool to generate a comprehensive reference. Identity threshold was kept default, i.e., 90%. DESeq2, an R package, was used for differential gene expression analysis between control and treated samples of both resistant and susceptible genotypes. Differentially expressed transcripts were selected based on a cutoff log2 fold change of 2 and a cutoff *p*-value of 0.05.

The clustered transcriptome was annotated using the DIAMOND BLASTX version 2.0.9.147^6^ tool against NCBI’s non-redundant protein database (NRDB),^7^ UniProt/SwissProt database,^8^ and plantTFDB^9^ with a cutoff *e*-value of ≤10^––5^. UniProt/SwissProt ID mapping functionality was used to get gene ontology (GO) and pathway annotation for transcripts. The Transeq utility from EMBOSS version 6.6.0^10^ package was used to convert the transcripts into the longest possible open reading frame. Orthologous groups of protein sequences were identified using a standalone version of emapper version 2.0.1 against eggNOG version 5.0.^11^ Protein sequences were classified into families and predicted domains, important sequence signatures using a standalone version of InterProScan 5.39--77.0.^12^ To study pathogen receptor genes, we mapped the transcripts on PRGDB^13^ manually curated reference protein sequences using the DIAMOND BLASTX utility with an *e*-value of ≤ 10^–5^.

### Validation of Differentially Expressed Genes Using Quantitative Realtime-PCR

Total RNA was isolated from leaves of two biological replicates at 5 dpi. The untreated plants were considered as a control in the present experiment. The total RNA was isolated with the RNeasy Plant Mini Kit (Qiagen, Germany) according to the manufacturer’s guidelines. To eliminate potential genomic DNA contamination, RNAs were treated with DNase I (Fermentas, Lithuania). RNAs were quantified using NanoDrop (ND1000), and the integrity of the isolated RNA was assessed on the formaldehyde-agarose gel (1%). First-strand cDNA was synthesized to reverse transcription reaction from 1 μg RNA using the RevertAid First Strand cDNA Synthesis Kit (Fermenats, Lithuania) following the manufacturer’s instructions. cDNAs were stored at –80°C until used in quantitative realtime-PCR (qRT-PCR).

Primers for selected PB-induced transcripts were designed using Primer-BLAST^14^ to amplify a region of 160–230 bp. Expression analyses of selected differentially expressed genes (DEGs) were performed in LightCycler^®^ 480 II instrument (Roche, Germany). A single 10 μl PCR mixture contained 1 × LightCycler^®^ 480 SYBR Green I master mix (Roche, Germany), 1 μl of cDNA, and 1 μM of each primer (10 μM). The PCR cycling was programmed as follows: initial denaturation at 95°C for 5 min, 45 cycles at 95°C for 10 s, 58°C for 10 s, and 72°C for 15 s. *AcActin* was used as a reference gene. The details of primers used in the present qPCR analyses are depicted in Supplementary Table 1. The analyses were performed using two biological replicates along with respective three technical replicates, and the relative fold change in transcript concentration was measured according to the 2^–ΔΔCT^ method (Livak and Schmittgen, 2001).

### Biochemical Analyses

#### Anti-oxidative and Defense Enzyme Assay

Leaf samples were harvested at 5 dpi from both control and infected plants of resistant and susceptible genotype for estimation of antioxidant enzyme activities of catalase (CAT), ascorbate peroxidase (APX), guaiacol peroxidase (GPX), superoxide dismutase (SOD), and phenylalanine lyase (PAL). Enzyme extract was prepared as per Roylawar and Kamble (2017) with modification. The sample was ground in liquid N_2_, and 200 mg of this sample was homogenized by adding chilled potassium phosphate buffer of 0.1 M and pH of 7.2 with Na-EDTA (0.1 M) and PVP (0.5%). The supernatant obtained after centrifugation (14,000*g*) at 4°C for 20 min was used for performing enzyme assays.

#### Catalase

Catalase was determined by the decomposition of H_2_O_2_, which was measured by recording the decrease in absorbance at 240 nm (Volk and Feierabend, 1989). For the determination of CAT activity, a 3 ml reaction volume was made up by adding the reaction medium containing 0.1 M potassium phosphate buffer (pH 7.0) and 30 mM H_2_O_2_ to the enzyme extract. One unit activity of CAT was determined by the amount of enzyme that used 1 μmol H_2_O_2_ per minute.

#### Ascorbate Peroxidase

The APX activity was assayed using a modified method of Nakano and Asada (1981). The 1 ml assay mixture contained 0.5 M Tris HCl buffer (pH 7.6), 0.1 mM Na_2_EDTA, 0.5 mM ascorbic acid, and 5 mM H_2_O_2_ along with enzyme extract. To initiate the reaction, H_2_O_2_ was added at last, and the absorbance at 290 nm was recorded for 3 min. The enzyme activity was calculated by the determination of reduction in ascorbate by using an extinction coefficient of 2.8 mM^–1^ cm^–1^ that was expressed in terms of millimole of ascorbate per minute per gram fresh weight.

#### Guaiacol Peroxidase

Peroxidase activity was assayed by using guaiacol as the substrate by following the method described by Xu et al. (2011). The assay system consisted of 0.1 M phosphate buffer pH 7.0, 30 mM guaiacol, 20 mM H_2_O_2_, and a suitable aliquot of enzyme in a final volume of 3 ml. The GPX activity was determined spectrophotometrically by measuring the increase in absorbance at 470 nm by the conversion of guaiacol to tetraguaiacol due to its oxidation. The molar extinction coefficient of tetraguaiacol was taken as 26.6 mM^–1^cm^–1^. One unit of enzyme activity is defined as the formation of 1 μmol product of tetraguaiacol by the enzyme catalyzing the reaction per minute at 30°C.

#### Superoxide Dismutase

The SOD activity was determined spectrophotometrically by measuring the ability of the enzyme to inhibit the photochemical reduction of nitro blue tetrazolium (NBT) (Beauchamp and Fridovich, 1971). The assay mixtures contained 50 mM phosphate buffer (pH 7.8), 60 μM riboflavin, 20 mM methionine, 1 mM EDTA, and 1 mM NBT together with enzyme extract. One unit of enzyme activity was taken as the amount of enzyme that caused the 50% inhibition of NBT reduction in the light which recorded a reduction in absorbance reading at 560 nm of up to 50% compared with tubes without enzyme.

#### Phenylalanine Lyase

The PAL activity was estimated by referring to the method of Khan and Vaidyanathan (1986), with modifications. The estimation of PAL activity was performed based on the rate of conversion of phenylalanine to cinnamate. A reaction mixture was made by adding 1.5 ml of 50 mM Tris-HCL buffer (pH 8.3), 0.3 ml of 1 mM L-phenylalanine, 0.9 ml distilled water, and 0.3 ml of enzyme extract. Furthermore, this reaction mixture was incubated in a water bath at 30°C for 60 min. The reaction was stopped by the addition of 1 ml of 2 N HCl. The absorbance of the solution was recorded at 290 nm using a UV spectrophotometer. A unit of enzyme activity is determined by the conversion of 1 μmol L-phenylalanine to cinnamic acid per minute.

### Statistical Analysis

The data of qRT-PCR and enzyme assays were analyzed using one-way ANOVA. Significant differences were analyzed using Duncan’s multiple range tests (*p* < 0.05). The analyses were performed using SPSS 16.0 and Microsoft excel. Figures were prepared using OriginPro 8.5.

## Results

### Symptoms After *Alternaria porri* Infection

**FIGURE 1:**
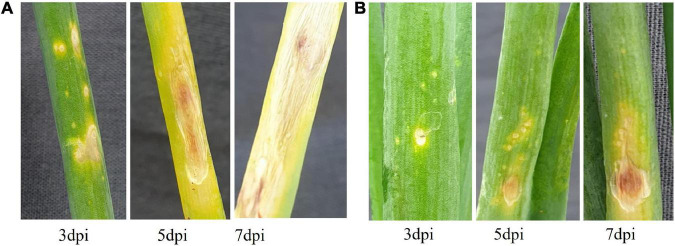
Purple blotch (PB) symptom development in onion after pathogen inoculation; **(A)** Agrifound rose (AFR) and **(B)** Arka Kalyan (AK).

Inoculation of *A. porri* spore solution on AK and AFR developed typical PB symptoms. In this study, the expected susceptible variety (AFR) developed numerous larger lesions than AK (Figure 1). The percent disease index (PDI) was evaluated at 3, 5, and 7 dpi in the greenhouse which was found higher in AFR compared with AK (Supplementary Figure 1). We sequenced the transcriptome at 5 dpi to study the differential molecular response to PB.

### RNA Sequencing Data and Gene Expression in Response to *Alternaria porri*

#### Reads and Assembly Stat

Paired-end RNA sequencing of control and inoculated plants of both varieties was performed in duplicate using the Illumina HiSeq 2500 platform, which yielded raw reads ranging from 46659974 to 119087128 (Supplementary Table 2). After data filtering (q30), 97%–99% of reads were survived, and these reads were further used for the construction of *de novo* assembly using Trinity. A final mega assembly comprised a total of 122,660 non-redundant transcripts. The maximum transcript length was 15,682 bases with an average length of 640 bases. N50 value of final assembled transcripts was 1,685 bases. The GC content of the present transcriptome of onion was 42.99% (Table 1). RNA sequencing raw reads were submitted to the NCBI SRA database under BioProject: PRJNA796147.

**TABLE 1:** Assembly statistics of onion transcriptome in response to purple blotch (PB).

Parameters	Mega-assembly
Total No. of transcripts	122660
Length of transcriptome (Mb)	78536108
Max transcript length (bases)	15682
Average transcript length (bases)	640.27
Median transcript length (bases)	286
N50 length (bases)	1685
% GC	42.99

#### Differential Gene Expression

Differential gene expression was compared between the *A. porri* infected and control leaf samples of both resistant and susceptible onion genotypes at 5 dpi. In susceptible genotype AFR, 8,064 genes were upregulated, and 248 genes were downregulated, whereas in resistant genotype AK, 832 transcripts were upregulated, and 564 were downregulated on infection by *A. porri.* A large portion of transcripts was unknown due to the non-availability of well-annotated genomic resources in onion. The top 100 significant differentially expressed transcripts were schematically represented in the heat map (Supplementary Figure 2). Details of differentially expressed genes, such as fold change and functional annotation, are provided in Supplementary Material 1.

#### Functional Annotation

The differentially expressed transcripts were functionally annotated using gene ontology (GO) and orthologous groups (COG) enrichment analyses. Total 188 DEGs of AK were categorized to GO terms belonging to molecular function (MF) of which 103 were upregulated transcripts and 85 were downregulated, while in AFR, 202 were upregulated, and 27 downregulated DEGs were annotated as having MF. In AK, the top three GO terms in the MF category are oxidoreductase activity [GO:0016491], monooxygenase activity [GO:0004497], and metal ion binding [GO:0046872], whereas in AFR, oxidoreductase activity [GO:0016491], metal ion binding [GO:0046872], and chitinase activity [GO:0004568] were dominantly enriched. In the cellular component (CC) category, 49 transcripts were upregulated, and 65 were downregulated in AK, whereas 103 transcripts showed upregulation, and 12 showed downregulation in AFR. GO terms in the CC category such as an integral component of the membrane [GO:0016021], chloroplast thylakoid membrane [GO:0009535], and cell wall [GO:0005618], were dominantly enriched in AK. In AFR, an integral component of the membrane [GO:0016021], extracellular region [GO:0005576], and chloroplast thylakoid membrane [GO:0009535] were the top three GO terms upregulated in the CC category. The biological process (BP) category of GO annotation analyses revealed 57 upregulated and 64 downregulated GO terms in AK and 127 upregulated and 16 downregulated GO terms in AFR. GO terms, such as pectin catabolic process [GO:0045490], photorespiration [GO:0009853], and protein-chromophore linkage [GO:0018298], were overrepresented in BP category in AK, whereas GO terms, such as carbohydrate metabolic process [GO:0005975], cell wall macromolecule catabolic process [GO:0016998], and chitin catabolic process [GO:0006032], were highly enriched in AFR.

**FIGURE 2:**
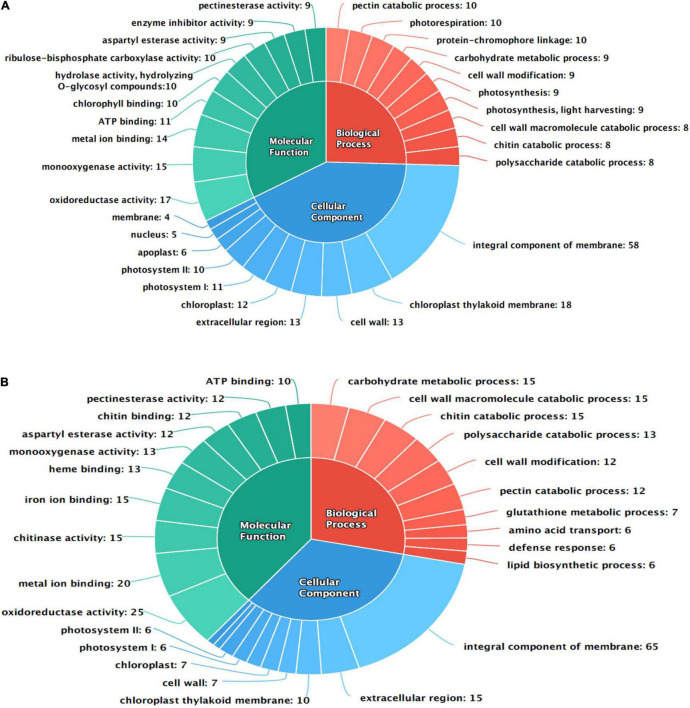
Functional annotation of differentially expressed transcripts in response to PB in onion using gene ontology (GO); **(A)** AK and **(B)** AFR.

Gene ontology terms involved in plant defense response were also overrepresented in the MF category such as hydrolase activity, hydrolyzing O-glycosyl compounds [GO:0004553], enzyme inhibitor activity [GO:0004857], and chitin-binding [GO:0008061]. In the BP category, cell wall modification [GO:0042545], glutathione metabolic process [GO:0006749], and defense response [GO:0006952] are also enriched in present data in response to PB disease in onion genotypes. The top ten GO terms in each category are shown in Figure 2.

**FIGURE 3:**
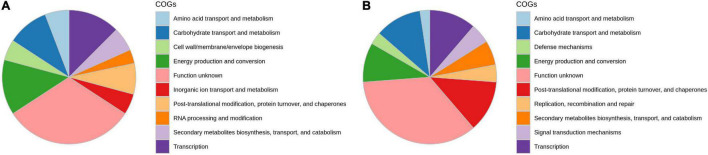
Functional annotation of differentially expressed transcripts in response to PB in onion using orthologous groups (COG) categories; **(A)** AK and **(B)** AFR.

The analyses of the distribution of clusters of COG in DEGs showed that the majority of genes are involved in energy production and conversion, transcription, carbohydrate transport and metabolism, posttranslational modification and protein turnover, signal transduction, cell wall biogenesis, and defense mechanism, which are involved in onion genotypes after *A. porri* infection (Figure 3). Kyoto Encyclopedia of Genes and Genomes (KEGG) pathway studies demonstrated that most DEGs are involved in glycan metabolism, carbohydrate degradation, lipid metabolism, and alkaloid biosynthesis pathways.

Transcripts of DEGs were blasted against TFDB, and several transcription factors were identified from the present *RNAseq* data. Among them, NAC, ERF, MYB, bHLH, MYB-related, and WRKYs were found to be dominant, and their differential expression in response to PB disease in onion reprogrammed the overall pattern of gene expression in disease state (Supplementary Figure 4).

Furthermore, DEGs were examined for species distribution and found that majority of annotated transcripts of AK were matched with *Elaeis guineensis* (23.05%), followed by *Asparagus officinalis* (9.38%) and *Allium cepa* (5.47%). In AFR, the majority of annotated transcripts showed homology with *E. guineensis* (21.32%) followed by *A. officinalis* (12.54%) and *Allium sativum* (5.64%). The top ten species’ distribution of DEGs in both genotypes is presented in Supplementary Figure 3.

Transcripts of both AK and AFR were blasted against PRGdb, and we found that 73 transcripts showed homology with reference pathogen receptor genes (PRGs) (Supplementary Figure 3). Different PRGs were differentially expressed in AK and AFR. Hm2, Serk3A, PBS1, Pid2, and Ve2 were top upregulated PRGs in AFR, while Serk3A, Mlo, PBS1, and Pid2 were top PRGs in AK. Details of PRGs with rgene-id are given in Supplementary Material 2 and Supplementary Figure 5.

### Defense-Related Differentially Expressed Genes in Response to Purple Blotch in Onion

#### Pathogenesis-Related Proteins in Response to *Alternaria porri*

A total of seven classes of pathogenesis-related (PR) proteins were found to be differentially expressed in onion genotypes with response to PB infection. Expression of *PR-1* (*Antifungal*), *PR-2* (β*-1,3-Glucanase*), *PR-3* (*Chitinases*), *PR-4* (*Chitinases types I, II*), *PR-5* (*Thaumatin-like*), *PR-9* (*Peroxidase*), and *PR-10* (*Ribonuclease-like*) was upregulated by several-fold on *A. porri* infection in onion. Transcripts for PR-1 were upregulated by 3.6–5.7-fold, PR-2 by 3.7–6.7-fold, PR-3 by 3–8-fold, PR-4 by 5.2–9.2-fold, PR-5 by 3.7-fold, PR-9 by 7.2–13.1-fold, and PR-10 by 4.7–8.1-fold. Among these, PR-5 was only upregulated in resistant genotype AK.

#### Receptor-Like Kinases in Response to *Alternaria porri*

*Receptor-like serine/threonine-protein kinase*, *protein kinase domain-containing protein*, *wall-associated receptor kinase-like*, *brassinosteroid LRR receptor kinase*, *CBL-interacting serine/threonine-protein kinase*, and *calcium-dependent protein kinase* were upregulated by 4.8, 3.8, 3.3, 7.8, 5.3, and 2.8 old, respectively, in AK. *Salt tolerance receptor-like cytoplasmic kinase* 1 (6.8-fold), *receptor-like serine/threonine-protein kinase* (6.7-fold), *protein kinase* domain-containing protein (4.1-fold), *brassinosteroid LRR receptor kinase* (6.2-fold), *CBL-interacting serine/threonine-protein kinase* (11.7-fold), and *calcium-dependent protein kinase* (3.4-fold) showed increased expression in AFR.

#### Genes for Phytohormones in Response to *Alternaria porri*

Genes involved in the phytohormone biosynthesis process were differentially expressed onion genotypes under PB infection. Transcripts for ethylene synthesis, such as *1-aminocyclopropane-1-carboxylic acid synthase* (*ACS*), and *1-aminocyclopropane-1-carboxylic acid oxidase* (*ACO*), were upregulated by 4.7–7.4-fold and 3.6–7.2-fold in present studies. Similarly, transcripts for key ABA biosynthetic gene *9-cis-epoxycarotenoid dioxygenase* (*NCED*) were upregulated by 4.6–6.2-fold in response to *A. porri* infection in onion. *Linoleate 9S-lipoxygenase*, a jasmonic acid marker gene, was upregulated 7.3-fold in AK and 4.5-fold in AFR. Furthermore, *12-oxophytodienoate reductase*, which is involved in jasmonic acid signaling and oxylipin biosynthesis, was overexpressed by 8.6-fold in AK and 3.5-fold in AFR. Transcripts for *Phospholipase A* also showed 5.3-fold increased expression in AK. Transcripts for *auxin-responsive protein* (−5- to −11-fold) and *auxin transporter* (−4.3-fold) were downregulated in AK, whereas *auxin-related protein 2* and *auxin-response protein* were found to be upregulated by 7.4- and 7.6-fold, respectively, in AFR. In addition, several ethylene response factors (ERFs) were also expressed differentially in onion in response to PB infection.

#### Cell Wall Integrity Genes in Response to *Alternaria porri*

Genes involved in cell wall integrity, such as *pectin methylesterase inhibitors* (*PMEI*) and *polygalacturonase inhibitor proteins* (*PGIP*s), were found to be differentially expressed in onion genotypes under biotic stress. PMEI and PGIP were upregulated by 4.6- and 9.8-fold, respectively, in AK, whereas in AFR, PGIP was upregulated by 10.8-fold. The expression of *xyloglucan endotransglucosylase/hydrolase* with a function in cell wall biogenesis [GO:0042546], cell wall organization [GO:0071555], and the xyloglucan metabolic process [GO:0010411] was only increased in AK. *Pectate lyase* with polygalacturonase activity [GO:0004650] and pectin-catabolic process [GO:0045490] was downregulated by −7.8-fold in AK, while it was upregulated by 4.7-fold in AFR. In addition, transcripts for *pectinesterase* were also showed a 5–12-fold increased expression in both the genotypes.

#### Cytochrome P450 Monooxygenases in Response to *Alternaria porri*

Several CYPs were differentially expressed in the present *RNAseq* dataset of onion under biotic stress imposed by PB. Transcript levels of *CYP81, CYP81E, CYP86A, CYP89A2, CYP71A1, CYP71A9, CYP736A12, CYP709B2, CYP79A*, and *CYP85A1* were found to be elevated. Among these, *CYP81* and *CYP85A1* were found only in AK, whereas *CYP86A* and *CYP81E* were found to be upregulated only in AFR.

#### Other Defense-Related Genes

*Glutathione S-transferase* is an important gene in plant defense in biotic and abiotic stress response, and transcripts for this gene were observed to be increased in AK by 3.2–9.9-fold and in AFR by 3.3–14.5-fold, respectively. Other important defense genes, namely, *E3 ubiquitin-protein ligase*, *hypersensitive-induced response protein 1*, and *BTB/POZ domain-containing protein*, were upregulated by 9. 1-, 3. 8-, 6.5-fold in AK and 7. 5-, 6. 1-, 4.8-fold in AFR, respectively. *GABA transporter1* (*GAT1*) and the *ankyrin repeat domain-containing protein* were highly upregulated by 11.8- and 6-fold only in AK. A few proteases and peptidases were also expressed differentially in onion on PB infection. Thiol protease, aspartic protease, carboxypeptidase, serine carboxypeptidase, and metacaspase were upregulated in both genotypes.

#### Transcription Factors in Response to *Alternaria porri*

A large number of transcription factors have been expressed differentially in onions in response to biotic stress imposed owing to PB infection. ERF, WRKY, MYB, and NAC are the major TFs that are upregulated in the current *RNAseq* dataset. They are known to play a key role in the plant defense response against abiotic and biotic stress elements by reprogramming the gene expression pattern in the cell. In this study, *ERF1, ERF2, ERF14, ERF96, NAC62, NAC42, NAC47*, and *NAC7* and several other defense-related transcription factors were found to be upregulated.

#### Metabolism Related Genes in Response to *Alternaria porri*

Transcripts involved in flavonoid biosynthesis were also expressed differentially on PB infection in onion. *Flavonoid glucosyltransferase* and *flavonoid 3′-hydroxylase* were upregulated by 4.1- and 4.5-fold, respectively, in AK, while *flavonoid 3′-hydroxylase* was upregulated 5.8-fold in AFR. *Anthocyanidin synthase* and *Chalcone synthase* were downregulated by −4.4 and −5.6-fold in AK and AFR, respectively.

Several transcripts for genes in carbohydrate metabolism were expressed differentially on *A. porri* infection in onion genotypes. *Glycosyltransferase* (8.2-fold), *sucrose synthase* (4.4-fold), and *xylose isomerase* (3.1-fold) were overexpressed in AK after PB infection. Similarly, in AFR, *glycosyltransferase* (11-fold), *sucrose phosphate synthase* (6.3-fold), *Fructokinase* 1 (4.1-fold), and *Invertase* (3.2-fold) showed upregulation due to *A. porri* infection. *Glyceraldehyde -3-phosphate dehydrogenase* was downregulated by 3.2–4.3-fold in both onion genotypes in this study. Transcript for *Beta-glucosidase 23* with functional annotation negative regulation of the defense response [GO:0031348] was upregulated in AFR.

Amino acid transporters also showed differential expression in onions in response to PB disease. Transcripts for amino acid transporter protein (6.5-fold) and amino acid transporter domain-containing proteins (4.7 to 8.5-fold) upregulated in AK. Similarly, amino acid transporter protein (6.7-fold) and amino acid transporter domain-containing proteins (6–10-fold) showed upregulation in AFR.

### Validation by Quantitative Realtime-PCR

**FIGURE 4:**
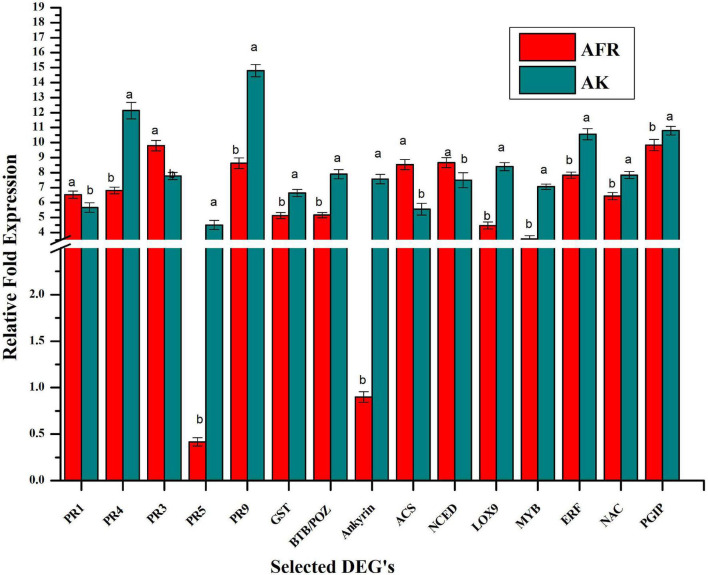
Validation selected differentially expressed genes by relative qPCR analysis in AK and AFR at 5 dpi. Data refer to the most representative of two independent repeated experiments. Each reaction was performed in triplicate, and values represent the average of these three technical replicates. Error bar showed the standard error of the mean, and different letters suggest statistically significant differences as per Duncan’s multiple range test (*p* < 0.05).

For validation of transcriptome, we selected 15 differentially expressed genes that play an important role in the plant defense response against diseases. These selected genes code for PR proteins and antioxidants, such as *PR1, PR3, PR4*, *PR5, and PR9*, and glutathione-S-transferase. Some of them are also involved in the synthesis of phytohormones, such as *ACS, NCED*, and *LOX*, and few of them code for transcription factors such as *MYB, ERF*, and *NAC.* Furthermore, genes for protein-containing *BTP/POZ domain, ankyrin repeat domain*, and *PGIP* were also validated using real-time qPCR (Figure 4). There was a good correlation (*R*^2^ = 0.87) between the levels of expression of genes in *RNAseq* and qPCR assays, which ascertain the reliability and quality of present transcriptome analysis (Supplementary Figure 6).

### Antioxidant Enzyme Assays

**FIGURE 5:**
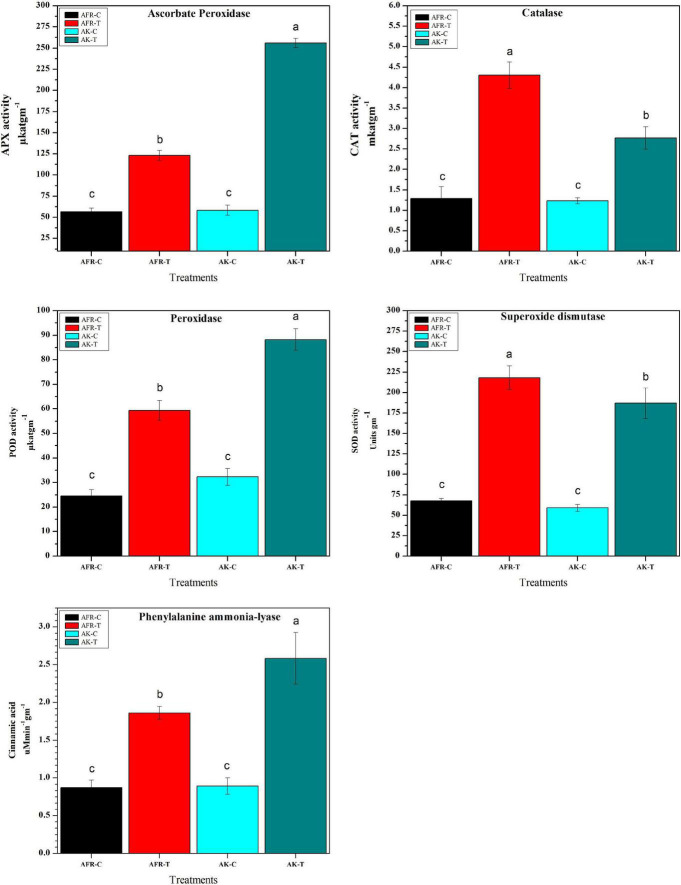
Effects of *Alternaria porri* infection on resistant and susceptible onion antioxidant and defense enzymes at 5 dpi. Data refer to the most representative of two independent repeated experiments. Values are expressed as the average of three biological replicates, each consisting of three plants pooled together. Error bar showed the standard error of the mean, and different letters suggest statistically significant differences as per Duncan’s multiple range test (*p* < 0.05).

Biochemical changes associated with PB infection were examined for the activity of antioxidant enzymes in the control and infected resistant and susceptible onion genotypes at 5 dpi. It was observed that infection with *A. porri* significantly increased the activity of all antioxidant enzymes examined. The activity of APX, GPX, and PAL was found significantly higher in the resistant genotype AK, whereas CAT and SOD activity was higher in the susceptible counterpart AFR. The catalase activity in infected AFR and AK was 2.8-fold and 2-fold higher than their respective controls, while the activity of ascorbate peroxidase was higher by 1.8 and 3.5-fold in AFR and AK, respectively. The guaiacol peroxidase activity was increased by 2.3-fold in AFR and 2.6-fold in AK after *A. porri* infection. The SOD activity was increased by 3.2-fold in AFR and 2.4-fold in AK after infection. The PAL assay showed a higher increase in activity in AK (2.4-fold) than in AFR (2.1-fold), while there is no significant change in activity that was observed in controls of both the genotypes (Figure 5).

## Discussion

Onion is an economically important crop in the world, and PB is one of the most devastating diseases of this crop. There is limited information available on the molecular response of onion to PB. *RNAseq* is one of the efficient advanced approaches for studying the molecular mechanism behind plant disease response. The main aim of this study is the transcriptome profiling of PB-resistant and susceptible genotypes.

### Pathogenesis-Related Proteins

Pathogenesis-related (PR) proteins were synthesized by a plant in adverse conditions owing to biotic or abiotic stress elements and are a vital component of plant defense response. They are involved in HR or SAR against pathogen infection and are regulated by the complex panoply of signaling pathways. They are classified into 17 families along with a putative new PR-18 group, which comprises fungus and SA-inducible carbohydrate oxidases (Custers et al., 2004; Jain and Khurana, 2018). In this investigation, PR proteins belonging to seven families (PR-1, PR-2, PR-3, PR-4, PR-5, PR-9, and PR-10) were showed upregulation in onion in response to PB disease. PR-4 proteins are comprised of chitinases and also have ribonuclease activity, and these accumulate in response to a pathogen attack or wounding. Bravo et al. (2003) demonstrated that the fungal elicitors, wound, exogenous ABA, and methyl jasmonate treatment resulted in the production of *ZmPR4* in maize. Recently, sugarcane PR-4 was reported to be involved in fungal cell death by virtue of ribonuclease, chitosanase, and chitinase action (Franco et al., 2019). Our previous study reported upregulation of chitinase gene in response to *Stemphylium* blight in onion caused due to a necrotrophic pathogen *Stemphylium vesicarium* (Roylawar et al., 2021). PR-5 (thaumatin-like) imparted fungal disease resistance in transgenic tobacco (Rajam et al., 2007). In addition, it has also been reported that PR1, PR2, PR4, and PR5 are induced in garlic after infection by *Fusarium* (Rout et al., 2016; Chand et al., 2017; Anisimova et al., 2021). PR-9 is comprised of peroxidase and is well known for its role in plant defense as a potent antioxidant in ROS scavenging. These peroxidases were upregulated in apples by the infection of *Alternaria* blotch caused by *Alternaria alternata* (Zhang et al., 2015). Thus, higher expression of PR proteins suggests the development of systemic acquired resistance against *A. porri* in onion.

### Transcription Factors

Few ERF transcription factors were upregulated in both genotypes under pathogen attack. ERF1 is known to be induced by phytohormones such as jasmonate and ethylene and imparts resistance against necrotrophic pathogens in *Arabidopsis* (Lorenzo et al., 2003; Berrocal-Lobo and Molina, 2004). Constitutive expression of *ERF1* and *ERF2* activates the pathogen-inducible plant defensin 1.2 (PDF1.2) gene (Maruyama et al., 2013). Similarly, the expression of JA/ET defense genes, such as *PDF1.2a, PR-3*, and *PR-4*, was upregulated by *ERF96* and positively regulates the resistance to necrotrophic fungi, which indicates its importance in ERF regulatory network (Catinot et al., 2015). *ERF14* is also known to play a key role in defense against *Fusarium oxysporum* in *Arabidopsis*, and the expression of other ERFs also depends on the expression of *ERF14.* Further *PDF1.2* and *Chitinase* expression levels were also increased in *ATERF14* overexpression lines, which suggests its prominent role in plant defense (Oñate-Sánchez et al., 2007). Thus, these ERFs might play a role *via* JA/ET signaling in PB disease response in onion. Several WRKY transcription factors were differentially expressed in response to *A. porri* infection in onion. *WRKY6* was reported to be a positive regulator for plant resistance to necrotrophic pathogen *via* JA, ET, ABA-induced gene expression, ROS accumulation, and upregulation of PR genes against *Phytophthora infestans* and *Ralstonia solanacearum* (Cai et al., 2015; Hong et al., 2018). Furthermore, *WRKY65* was reported to play a key role in imparting resistance against *Alternaria tenuissima* in herbaceous peony by regulating JA and SA levels (Wang et al., 2020). *MdWRKY75* was reported to increase the resistance to *A. alternata* in apples primarily by jasmonic acid signaling and antioxidant enzymes (Hou et al., 2021). These WRKY TFs are also upregulated in onion in response to PB infection and might be performing a similar function in onion also. Several NAC transcription factors were also upregulated in this study. *NAC* TFs were reported to be involved in the induction of plant defense against *Alternaria brassicicola* in *Arabidopsis* and *Brassica* (Saga et al., 2012; Mondal et al., 2020). NAC7 was reported to be a negative regulator of senescence and stay green trait in maize. RNAi lines of maize exhibited delayed senescence as well as an increase in total biomass (Mondal et al., 2020). In this study, NAC7 was highly upregulated in susceptible genotype (AFR) which might be due to higher senescence of leaves on PB infection. Similarly, several other transcription factors, such as MYB, bZIP, and C2H2, were also differentially expressed under *A. porri* infection in onion genotypes under study. They showed differential expression in other crops also in response to disease and plays important role in plant immunity (Verma et al., 2017; Yuan et al., 2019).

### Phytohormones

Phytohormones, such as jasmonic acid, salicylic acid, abscisic acid, and ethylene, play a key role in plant responses to biotic and abiotic stresses. In this study, genes in jasmonic acid and ethylene biosynthesis were highly upregulated in response to *A. porri* infection. JA and ET are mainly involved in defense against necrotrophic pathogens, while SA acts against biotrophic pathogens (Dong, 1998; Glazebrook, 2005; Zhang et al., 2018). *Acyl-CoA oxidase, 12-oxophytodienoate reductase, lipoxygenase*, and *3-ketoacyl-CoA thiolase* transcripts in jasmonic acid biosynthesis were also reported to be upregulated in response to other fungal diseases such as Bakanae disease in rice (Matić et al., 2016), *Fusarium* head blight of wheat (Pan et al., 2018), *Fusarium* wilt of Flax (Galindo-González and Deyholos, 2016), and *Sclerotium* stem rot in peanut (Bosamia et al., 2020). Ethylene is also an important phytohormone that acts hand in hand with JA to fight necrotrophic diseases. Key transcripts in ethylene biosynthesis, namely, ACS and ACO, were highly expressed in onion in response to *A. porri* infection. Transgenic rice with inducible ACS expression exhibited enhanced resistance to *Magnaporthe oryzae* and *Rhizoctonia solani* (Helliwell et al., 2013). Further *RNAseq* studies also found the upregulation of ET synthetic genes in response to the necrotrophic fungal pathogen (De Cremer et al., 2013; Matić et al., 2016; Pan et al., 2018). JA and ET modulate the expression of PR genes through a signaling network comprising various transcription factors and metabolites (Pieterse et al., 2012). *NCED*, a key gene in ABA biosynthesis, was also upregulated in onion genotypes due to infection of *A. porri.* ABA is known to regulate the various processes in plant-pathogen interactions (Fan et al., 2009). It plays an effective role in imparting disease resistance in plants against necrotrophic fungi (Ton and Mauch-Mani, 2004; Boba et al., 2020). PR1 and GST are the marker genes for the salicylic acid and were reported to be induced in the present dataset with response to PB in onion. GST’s main role is already known as the removal of toxic compounds and acts as an antioxidant. Upregulation of GST was reported in several plants after infection by necrotrophic pathogens such as *Alternaria brassicicola* (Schenk et al., 2000).

### Cell Wall Integrity Genes

The cell wall is the first physical barrier to the pathogen, and numerous changes occur in the cell wall in response to the pathogen (Malinovsky et al., 2014). Necrotrophic fungal pathogens degrade the cell wall matrix with the help of the secretion of different enzymes. The plant maintains cell wall integrity by inhibiting the cell wall degrading enzymes by the expression of different enzymes such as PMEI and PGIPs. The genes for these enzymes were highly upregulated in onion in response to PB pathogen. In *Arabidopsis*, it was found that PMEI helps in maintaining cell wall integrity by inhibiting the pectin methylesterase which imparted resistance against botrytis (Lionetti et al., 2017). PGIPs protect the cell wall from pathogen, and insects attack by inhibiting the depolymerization of pectin in the cell wall by polygalacturonases (Kalunke et al., 2015). PGIPs not only protect pectin from degradation but also lead to the production of longer oligogalacturonides that can be recognized as damage-associated molecular patterns that ultimately activate PTI and slow down the colonization of pathogen (Federici et al., 2006). These genes might also contribute toward defense response against PB in onion.

### Defense-Related Genes Exclusively Expressed in Arka Kalyan

A few genes were exclusively showed upregulation in a resistant genotype (AK), PR5, ankyrin repeat domains, GABA transporter, *CYP85A1*, etc. PR5 was reported to induce phytohormones and biotic and abiotic stresses in garlic. Its ectopic expression in *Arabidopsis* increased resistance to *Botrytis* by constitutive expression of defense-related genes, which suggests its broad role in plant defense (Rout et al., 2016). Similarly, ankyrin repeat domains are widely distributed and well-studied protein–protein interaction domains in several processes in plants including response biotic and abiotic stresses. In rice, ankyrin repeat-containing protein involved in providing defense against *Magnaporthe oryzae*, and overexpression lines showed higher expression of SA and JA responsive genes (Mou et al., 2013). Similarly, the expression of ankyrin repeat protein, *GmARP1*, imparted resistance in transgenic soybean against *Fusarium virguliforme* (Ngaki et al., 2016). CYPs are known to play an important role in biotic and abiotic stress response in plants by modulating levels of antioxidant molecules, phytohormones, and other metabolites (Pandian et al., 2020). *CYP85A1* is reported to be involved in brassinosteroid biosynthesis, and its overexpression in tobacco increased tolerance to *Phytophthora nicotianae* in transgenic plants by elevating brassinosteroid level and modulation of phytohormone and defense enzyme activities (Duan and Song, 2019). This suggests that *CYP85A1* might play a role in defense response against pathogen. GABA accumulation after abiotic or biotic stress is reported in several plants (Rashmi et al., 2018), and it might help in enhancing host immunity against fungal pathogens by modulating oxidative enzymes (Shelp et al., 2021). GABA transporter 1 (*GAT1*) involved in GABA influx into the cell (Batushansky et al., 2015). It suggests GABA’s important role in plant defense response.

### Metabolism-Related Genes in Response to *Alternaria porri* Infection

Secondary metabolites play an important role in plant defense against biotic and abiotic stresses by their antioxidant and antibacterial properties. Onions are a rich source of secondary metabolites such as flavonoids (Khandagale and Gawande, 2019). *Flavonoid glucosyl-transferase* is involved in the glycosylation of these flavonoids. They are known to regulate quercetin and kaempferol levels in plants and govern the plant defense against the pathogen (Campos et al., 2019). *Flavonoid 3′-hydroxylase*, another gene in the flavonoid pathway, was reported to be involved in the fungal pathogen-induced production of phytoalexins (Boddu et al., 2004). Glycosyltransferase is one of the players in JA-SA cross-talk during defense response signaling in plants. The overexpression of *UGT76B1* in *Arabidopsis* increased the resistance against a necrotrophic pathogen; *Alternaria brassicicola* also decreased the senescence with upregulation of JA dependent pathway. On the contrary, silencing of this gene led to reduced tolerance to *A. brassicicola*, induction of SA pathway, and elevated resistance to the biotrophic pathogen (von Saint Paul et al., 2011). Sugars and carbohydrates are the prime sources of energy. Several carbohydrate metabolisms and energy production genes showed differential expression in this study such as sucrose synthase, sucrose phosphate synthase, and invertase. These are reported to play role in defense response by interaction with phytohormones signaling in plants (Morkunas and Ratajczak, 2014; Tauzin and Giardina, 2014). The upregulation of these genes suggests that they might be playing such a role in defense response against PB in onion as well.

### Pathogen Receptor Genes From PRGdb

PRGdb is a database of PRGs that provide constant updates on plant resistance genes (Calle García et al., 2021). In this analysis, we found 73 transcripts having homology with reference PRGs in PRGdb. *Hm1 and Hm2* code for the NADPH-dependent HC-toxin reductases which were reported to protect maize from the toxin produced by a fungal pathogen (Dehury et al., 2014). Serk3a belongs to somatic embryogenesis receptor kinases and is involved in defense response. Loss of function mutants for Serk3a was unable to induce defense response against *P. infestans* in tobacco (Chaparro-Garcia et al., 2011) and also did not exhibit any defense response in potato after oligopeptide Pep-13 treatment (Nietzschmann et al., 2019). PBS1 is a member of receptor-like cytoplasmic kinases and is involved in the pattern triggered defense response in plants (Swiderski and Innes, 2001; Tang et al., 2017). Pid2 is a transmembrane receptor-like kinase that plays role in a rice blast resistance (Sharma et al., 2016). It was reported that E3 ubiquitin ligase and Pid2 interaction involved in the regulation of cell death and immunity against rice blast disease (Wang et al., 2015). Another PRG, Ve2, was found to impart resistance to wilt caused by *Verticillium* (Chen et al., 2017). These pathogen receptor genes might also play a key role in imparting immunity against PB in onion. These PRGs need to be investigated further for a better understanding of plant-pathogen interaction in onion.

### Antioxidant and Defense Enzymes

Biotic stress led to the generation of ROS in plants which help to fight pathogen *via* HR, and PCD (Apel and Hirt, 2004; Roylawar et al., 2021) and ROS also act as secondary messengers in signaling defense response (Yan et al., 2007; Hasanuzzaman et al., 2020). In this study, activities of antioxidant and defense enzymes, such as superoxide dismutase, catalase, ascorbate peroxidase, guaiacol peroxidase, and phenyl ammonia lyse, were increased in *A. porri* infected onion genotypes over control plants. GPX protects cells by neutralizing ROS radicals and functions as an antifungal agent in plant disease response (Chavan et al., 2013; Pieczul et al., 2020). GPX is also involved in lignin synthesis which acts as a barrier to pathogen spread in infected tissues (Tayefi-Nasrabadi et al., 2011; Roylawar et al., 2021). APX and CAT both play important roles in scavenging hydrogen peroxide. CAT plays a key role in the detoxification of H_2_O_2_ generated in peroxisome during pathogen attacks (Meena et al., 2016; Roylawar et al., 2021). Excessive H_2_O_2_ is scavenged by the ascorbate-glutathione cycle involving APX and was reported that APX activity, as well as gene expression level, gets elevated during pathogen infection (Meena et al., 2016). SOD performs dismutation of highly toxic oxygen radicals to oxygen and comparatively less toxic hydrogen peroxide. SOD activity is often increased in various biotic and abiotic stress situations, and higher activity is correlated with the resistance to the oxidative stress caused by abiotic stress as well as pathogens (Ehsani-Moghaddam et al., 2006; Gill and Tuteja, 2010; Youssef et al., 2020). These enzyme activities were also reported to be modulated by phytohormones (Szöke et al., 2021). Thus, in this study, in onion, reprograming of these genes led to the activation of antioxidant enzymes in response to PB.

## Conclusion

The present *RNAseq* analysis discovered several DEGs in onion in response to PB disease. Functional annotation analysis by GO and COG revealed that a large number of genes in several BPs including defense-related terms were enriched. This suggests that a large number of genes play role in PB response in onions. Several pathogen recognition genes were also discovered from PRGdb analysis. Defense-related genes, such as PR proteins, antioxidants, phytohormones biosynthesis and signaling, cell wall integrity, and transcription factors, were found to be induced in PB disease in onion. Antioxidant enzymes were found to play a key role in PB resistance in onion. Further investigation is required to identify key candidate genes necessary for imparting PB resistance using genetic engineering. This is the first report of transcriptome analysis of onion in response to PB, and thus, data generated in the present investigation will help researchers for further research in PB-onion interaction.

## Data Availability Statement

The datasets presented in this study can be found in online repositories. The names of the repository/repositories and accession number(s) can be found below: National Center for Biotechnology Information (NCBI) BioProject database under accession number PRJNA796147.

## Author Contributions

KK and SG conceived idea and designed the study. KK and PR performed experiment and statistical analysis. OK, AK, and KK contributed in bioinformatics analysis and visualizations. PK assisted in performing the experiment. AA, MS, and SG provided resources. KK, OK, PR, and PK wrote the manuscript. All authors played role in revising manuscript and approved the final version.

## Conflict of Interest

The authors declare that the research was conducted in the absence of any commercial or financial relationships that could be construed as a potential conflict of interest.

## Publisher’s Note

All claims expressed in this article are solely those of the authors and do not necessarily represent those of their affiliated organizations, or those of the publisher, the editors and the reviewers. Any product that may be evaluated in this article, or claim that may be made by its manufacturer, is not guaranteed or endorsed by the publisher.

## Footnotes

1
https://www.fao.org/faostat/en/#data/QCL

2
https://www.bioinformatics.babraham.ac.uk/projects/fastqc/

3
https://github.com/marcelm/cutadapt/

4
https://github.com/trinityrnaseq/trinityrnaseq/wiki

5
http://weizhong-lab.ucsd.edu/cd-hit/download.php

6
https://ab.inf.uni-tuebingen.de/software/diamond

7
http://ftp.ncbi.nlm.nih.gov/blast/db/

8
https://www.uniprot.org/

9
http://planttfdb.cbi.pku.edu.cn/

10
http://emboss.sourceforge.net/download/

11
http://eggnogdb.embl.de/#/app/downloads

12
https://www.ebi.ac.uk/interpro/download.html

13
http://prgdb.org/prgdb4

14
https://www.ncbi.nlm.nih.gov/tools/primer-blast/
