# Supplementary material for: Comparative Transcriptome Analysis of Onion in Response to Infection by Alternaria porri (Ellis) Cifferi
Source: Front Plant Sci. 2022 Apr 11;13:857306. doi: 10.3389/fpls.2022.857306 (PMC9036366; doi:10.3389/fpls.2022.857306)
Supplement: Supplementary file 3 [file Data_Sheet_3.ZIP › stat_enzyme assays/Ascorbate.pdf]

```
ONEWAY VAR00002 BY VAR00001
  /STATISTICS DESCRIPTIVES HOMOGENEITY
  /MISSING ANALYSIS
  /POSTHOC=TUKEY DUNCAN LSD ALPHA(0.05).
```

Oneway

Notes

Output Created		13-Feb-2022 17:50:10
Comments		
Input	Active Dataset	DataSet0
	Filter	<none>
	Weight	<none>
	Split File	<none>
	N of Rows in Working Data File	12
Missing Value Handling	Definition of Missing	User-defined missing values are treated as missing.
	Cases Used	Statistics for each analysis are based on cases with no missing data for any variable in the analysis.
Syntax		ONEWAY VAR00002 BY VAR00001 /STATISTICS DESCRIPTIVES HOMOGENEITY /MISSING ANALYSIS /POSTHOC=TUKEY DUNCAN LSD ALPHA(0.05).
Resources	Processor Time	00:00:00.047
	Elapsed Time	00:00:00.033

```
[DataSet0]
```

Descriptives

VAR00002

	N	Mean	Std. Deviation	Std. Error	95% Confidence Interval for Mean Lower Bound	95% Confidence Interval for Mean Upper Bound	Minimum	Maximum
1	3	56.6967	7.35469	4.24623	38.4266	74.9667	48.39	62.38
2	3	1.2299E2	10.18676	5.88133	97.6847	148.2953	112.24	132.50

Descriptives

VAR00002

	N	Mean	Std. Deviation	Std. Error	95% Confidence Interval for Mean		Minimum	Maximum
					Lower Bound	Upper Bound		
3	3	58.3633	10.20065	5.88935	33.0235	83.7032	48.23	68.63
4	3	2.5602E2	9.42148	5.43949	232.6124	279.4209	246.40	265.23
Total	12	1.2352E2	85.01366	24.54133	69.5016	177.5318	48.23	265.23

Test of Homogeneity of Variances

VAR00002

Levene Statistic	df1	df2	Sig.
.063	3	8	.978

ANOVA

VAR00002

	Sum of Squares	df	Mean Square	F	Sig.
Between Groups	78799.190	3	26266.397	299.606	.000
Within Groups	701.358	8	87.670		
Total	79500.548	11			

Post Hoc Tests

Multiple Comparisons

Dependent Variable:VAR00002

	(I) VAR00001	(J) VAR00001	Mean Difference (I-J)	Std. Error	Sig.	95% Confidence Interval	
						Lower Bound	Upper Bound
Tukey HSD	1	2	-66.29333*	7.64503	.000	-90.7754	-41.8112
		3	-1.66667	7.64503	.996	-26.1488	22.8154
		4	-199.32000*	7.64503	.000	-223.8021	-174.8379
	2	1	66.29333*	7.64503	.000	41.8112	90.7754
		3	64.62667*	7.64503	.000	40.1446	89.1088
		4	-133.02667*	7.64503	.000	-157.5088	-108.5446
	3	1	1.66667	7.64503	.996	-22.8154	26.1488
		2	-64.62667*	7.64503	.000	-89.1088	-40.1446
		4	-197.65333*	7.64503	.000	-222.1354	-173.1712
	4	1	199.32000*	7.64503	.000	174.8379	223.8021

*. The mean difference is significant at the 0.05 level.

Multiple Comparisons

Dependent Variable:VAR00002

	(I) VAR00001	(J) VAR00001	Mean Difference (I-J)	Std. Error	Sig.	95% Confidence Interval	
						Lower Bound	Upper Bound
Tukey HSD	4	2	133.02667*	7.64503	.000	108.5446	157.5088
		3	197.65333*	7.64503	.000	173.1712	222.1354
LSD	1	2	-66.29333*	7.64503	.000	-83.9228	-48.6639
		3	-1.66667	7.64503	.833	-19.2961	15.9628
		4	-199.32000*	7.64503	.000	-216.9495	-181.6905
	2	1	66.29333*	7.64503	.000	48.6639	83.9228
		3	64.62667*	7.64503	.000	46.9972	82.2561
		4	-133.02667*	7.64503	.000	-150.6561	-115.3972
	3	1	1.66667	7.64503	.833	-15.9628	19.2961
		2	-64.62667*	7.64503	.000	-82.2561	-46.9972
		4	-197.65333*	7.64503	.000	-215.2828	-180.0239
	4	1	199.32000*	7.64503	.000	181.6905	216.9495
		2	133.02667*	7.64503	.000	115.3972	150.6561
		3	197.65333*	7.64503	.000	180.0239	215.2828

*. The mean difference is significant at the 0.05 level.

Homogeneous Subsets

VAR00002

	VAR00001	N	Subset for alpha = 0.05		
			1	2	3
Tukey HSD[a]	1	3	56.6967		
	3	3	58.3633		
	2	3		1.2299E2	
	4	3			2.5602E2
	Sig.		.996	1.000	1.000
Duncan[a]	1	3	56.6967		
	3	3	58.3633		
	2	3		1.2299E2	
	4	3			2.5602E2
	Sig.		.833	1.000	1.000

Means for groups in homogeneous subsets are displayed.

a. Uses Harmonic Mean Sample Size = 3.000.

```
ONEWAY VAR00002 BY VAR00001
  /STATISTICS DESCRIPTIVES HOMOGENEITY
  /MISSING ANALYSIS
  /POSTHOC=TUKEY DUNCAN LSD ALPHA(0.05).
```

Oneway

Notes

Output Created		13-Feb-2022 17:51:47
Comments		
Input	Active Dataset	DataSet0
	Filter	<none>
	Weight	<none>
	Split File	<none>
	N of Rows in Working Data File	12
Missing Value Handling	Definition of Missing	User-defined missing values are treated as missing.
	Cases Used	Statistics for each analysis are based on cases with no missing data for any variable in the analysis.
Syntax		ONEWAY VAR00002 BY VAR00001 /STATISTICS DESCRIPTIVES HOMOGENEITY /MISSING ANALYSIS /POSTHOC=TUKEY DUNCAN LSD ALPHA(0.05).
Resources	Processor Time	00:00:00.031
	Elapsed Time	00:00:00.015

```
[DataSet0]
```

Descriptives

VAR00002

	N	Mean	Std. Deviation	Std. Error	95% Confidence Interval for Mean		Minimum	Maximum
					Lower Bound	Upper Bound		
1	3	56.6967	7.35469	4.24623	38.4266	74.9667	48.39	62.38
2	3	1.2299E2	10.18676	5.88133	97.6847	148.2953	112.24	132.50
3	3	58.3633	10.20065	5.88935	33.0235	83.7032	48.23	68.63

Descriptives

VAR00002

	N	Mean	Std. Deviation	Std. Error	95% Confidence Interval for Mean		Minimum	Maximum
					Lower Bound	Upper Bound		
4	3	2.5602E2	9.42148	5.43949	232.6124	279.4209	246.40	265.23
Total	12	1.2352E2	85.01366	24.54133	69.5016	177.5318	48.23	265.23

Test of Homogeneity of Variances

VAR00002

Levene Statistic	df1	df2	Sig.
.063	3	8	.978

ANOVA

VAR00002

	Sum of Squares	df	Mean Square	F	Sig.
Between Groups	78799.190	3	26266.397	299.606	.000
Within Groups	701.358	8	87.670		
Total	79500.548	11			

Post Hoc Tests

Multiple Comparisons

Dependent Variable:VAR00002

	(I) VAR00001	(J) VAR00001	Mean Difference (I-J)	Std. Error	Sig.	95% Confidence Interval	
						Lower Bound	Upper Bound
Tukey HSD	1	2	-66.29333*	7.64503	.000	-90.7754	-41.8112
		3	-1.66667	7.64503	.996	-26.1488	22.8154
		4	-199.32000*	7.64503	.000	-223.8021	-174.8379
	2	1	66.29333*	7.64503	.000	41.8112	90.7754
		3	64.62667*	7.64503	.000	40.1446	89.1088
		4	-133.02667*	7.64503	.000	-157.5088	-108.5446
	3	1	1.66667	7.64503	.996	-22.8154	26.1488
		2	-64.62667*	7.64503	.000	-89.1088	-40.1446
		4	-197.65333*	7.64503	.000	-222.1354	-173.1712
	4	1	199.32000*	7.64503	.000	174.8379	223.8021
		2	133.02667*	7.64503	.000	108.5446	157.5088

*. The mean difference is significant at the 0.05 level.

Multiple Comparisons

Dependent Variable:VAR00002

	(I) VAR00001	(J) VAR00001	Mean Difference (I-J)	Std. Error	Sig.	95% Confidence Interval Lower Bound	Upper Bound
Tukey HSD	4	3	197.65333*	7.64503	.000	173.1712	222.1354
LSD	1	2	-66.29333*	7.64503	.000	-83.9228	-48.6639
		3	-1.66667	7.64503	.833	-19.2961	15.9628
		4	-199.32000*	7.64503	.000	-216.9495	-181.6905
	2	1	66.29333*	7.64503	.000	48.6639	83.9228
		3	64.62667*	7.64503	.000	46.9972	82.2561
		4	-133.02667*	7.64503	.000	-150.6561	-115.3972
	3	1	1.66667	7.64503	.833	-15.9628	19.2961
		2	-64.62667*	7.64503	.000	-82.2561	-46.9972
		4	-197.65333*	7.64503	.000	-215.2828	-180.0239
	4	1	199.32000*	7.64503	.000	181.6905	216.9495
		2	133.02667*	7.64503	.000	115.3972	150.6561
		3	197.65333*	7.64503	.000	180.0239	215.2828

*. The mean difference is significant at the 0.05 level.

Homogeneous Subsets

VAR00002

	VAR00001	N	Subset for alpha = 0.05: 1	2	3
Tukey HSD[a]	1	3	56.6967		
	3	3	58.3633		
	2	3		1.2299E2	
	4	3			2.5602E2
	Sig.		.996	1.000	1.000
Duncan[a]	1	3	56.6967		
	3	3	58.3633		
	2	3		1.2299E2	
	4	3			2.5602E2
	Sig.		.833	1.000	1.000

Means for groups in homogeneous subsets are displayed.

a. Uses Harmonic Mean Sample Size = 3.000.
