# Supplementary material for: Comparative Transcriptome Analysis of Onion in Response to Infection by Alternaria porri (Ellis) Cifferi
Source: Front Plant Sci. 2022 Apr 11;13:857306. doi: 10.3389/fpls.2022.857306 (PMC9036366; doi:10.3389/fpls.2022.857306)
Supplement: Supplementary file 3 [file Data_Sheet_3.ZIP › stat_enzyme assays/Peroxidase.pdf]

```
ONEWAY VAR00002 BY VAR00001
  /STATISTICS DESCRIPTIVES HOMOGENEITY
  /MISSING ANALYSIS
  /POSTHOC=TUKEY DUNCAN LSD ALPHA(0.05).
```

Oneway

Notes

Output Created		13-Feb-2022 18:00:58
Comments		
Input	Active Dataset	DataSet0
	Filter	<none>
	Weight	<none>
	Split File	<none>
	N of Rows in Working Data File	12
Missing Value Handling	Definition of Missing	User-defined missing values are treated as missing.
	Cases Used	Statistics for each analysis are based on cases with no missing data for any variable in the analysis.
Syntax		ONEWAY VAR00002 BY VAR00001 /STATISTICS DESCRIPTIVES HOMOGENEITY /MISSING ANALYSIS /POSTHOC=TUKEY DUNCAN LSD ALPHA(0.05).
Resources	Processor Time	00:00:00.063
	Elapsed Time	00:00:00.032

```
[DataSet0]
```

Descriptives

VAR00002

	N	Mean	Std. Deviation	Std. Error	95% Confidence Interval for Mean		Minimum	Maximum
					Lower Bound	Upper Bound		
1	3	24.5867	4.22418	2.43883	14.0932	35.0801	21.20	29.32
2	3	59.3533	6.98911	4.03516	41.9914	76.7152	52.23	66.20
3	3	32.2733	5.94004	3.42949	17.5174	47.0292	26.32	38.20

Descriptives

VAR00002

	N	Mean	Std. Deviation	Std. Error	95% Confidence Interval for Mean		Minimum	Maximum
					Lower Bound	Upper Bound		
4	3	88.2533	7.58694	4.38032	69.4063	107.1003	81.23	96.30
Total	12	51.1167	26.69139	7.70514	34.1578	68.0756	21.20	96.30

Test of Homogeneity of Variances

VAR00002

Levene Statistic	df1	df2	Sig.
.250	3	8	.859

ANOVA

VAR00002

	Sum of Squares	df	Mean Square	F	Sig.
Between Groups	7517.660	3	2505.887	62.829	.000
Within Groups	319.074	8	39.884		
Total	7836.735	11			

Post Hoc Tests

Multiple Comparisons

Dependent Variable:VAR00002

	(I) VAR00001	(J) VAR00001	Mean Difference (I-J)	Std. Error	Sig.	95% Confidence Interval	
						Lower Bound	Upper Bound
Tukey HSD	1	2	-34.76667*	5.15650	.001	-51.2796	-18.2537
		3	-7.68667	5.15650	.485	-24.1996	8.8263
		4	-63.66667*	5.15650	.000	-80.1796	-47.1537
	2	1	34.76667*	5.15650	.001	18.2537	51.2796
		3	27.08000*	5.15650	.003	10.5671	43.5929
		4	-28.90000*	5.15650	.002	-45.4129	-12.3871
	3	1	7.68667	5.15650	.485	-8.8263	24.1996
		2	-27.08000*	5.15650	.003	-43.5929	-10.5671
		4	-55.98000*	5.15650	.000	-72.4929	-39.4671
	4	1	63.66667*	5.15650	.000	47.1537	80.1796
		2	28.90000*	5.15650	.002	12.3871	45.4129

*. The mean difference is significant at the 0.05 level.

Multiple Comparisons

Dependent Variable:VAR00002

	(I) VAR00001	(J) VAR00001	Mean Difference (I-J)	Std. Error	Sig.	95% Confidence Interval	
						Lower Bound	Upper Bound
Tukey HSD	4	3	55.98000*	5.15650	.000	39.4671	72.4929
LSD	1	2	-34.76667*	5.15650	.000	-46.6576	-22.8757
		3	-7.68667	5.15650	.174	-19.5776	4.2043
		4	-63.66667*	5.15650	.000	-75.5576	-51.7757
	2	1	34.76667*	5.15650	.000	22.8757	46.6576
		3	27.08000*	5.15650	.001	15.1891	38.9709
		4	-28.90000*	5.15650	.001	-40.7909	-17.0091
	3	1	7.68667	5.15650	.174	-4.2043	19.5776
		2	-27.08000*	5.15650	.001	-38.9709	-15.1891
		4	-55.98000*	5.15650	.000	-67.8709	-44.0891
	4	1	63.66667*	5.15650	.000	51.7757	75.5576
		2	28.90000*	5.15650	.001	17.0091	40.7909
		3	55.98000*	5.15650	.000	44.0891	67.8709

*. The mean difference is significant at the 0.05 level.

Homogeneous Subsets

VAR00002

	VAR00001	N	Subset for alpha = 0.05		
			1	2	3
Tukey HSD[a]	1	3	24.5867		
	3	3	32.2733		
	2	3		59.3533	
	4	3			88.2533
	Sig.		.485	1.000	1.000
Duncan[a]	1	3	24.5867		
	3	3	32.2733		
	2	3		59.3533	
	4	3			88.2533
	Sig.		.174	1.000	1.000

Means for groups in homogeneous subsets are displayed.

a. Uses Harmonic Mean Sample Size = 3.000.
