# Supplementary material for: Comparative Transcriptome Analysis of Onion in Response to Infection by Alternaria porri (Ellis) Cifferi
Source: Front Plant Sci. 2022 Apr 11;13:857306. doi: 10.3389/fpls.2022.857306 (PMC9036366; doi:10.3389/fpls.2022.857306)
Supplement: Supplementary file 3 [file Data_Sheet_3.ZIP › stat_enzyme assays/SOD.pdf]

```
ONEWAY VAR00002 BY VAR00001
  /STATISTICS DESCRIPTIVES HOMOGENEITY
  /MISSING ANALYSIS
  /POSTHOC=TUKEY DUNCAN LSD ALPHA(0.05).
```

Oneway

Notes

Output Created		13-Feb-2022 18:24:44
Comments		
Input	Active Dataset	DataSet0
	Filter	<none>
	Weight	<none>
	Split File	<none>
	N of Rows in Working Data File	12
Missing Value Handling	Definition of Missing	User-defined missing values are treated as missing.
	Cases Used	Statistics for each analysis are based on cases with no missing data for any variable in the analysis.
Syntax		ONEWAY VAR00002 BY VAR00001 /STATISTICS DESCRIPTIVES HOMOGENEITY /MISSING ANALYSIS /POSTHOC=TUKEY DUNCAN LSD ALPHA(0.05).
Resources	Processor Time	00:00:00.031
	Elapsed Time	00:00:00.026

```
[DataSet0]
```

Descriptives

VAR00002

	N	Mean	Std. Deviation	Std. Error	95% Confidence Interval for Mean		Minimum	Maximum
					Lower Bound	Upper Bound		
1	3	67.6267	5.03220	2.90534	55.1260	80.1273	62.32	72.33
2	3	2.1816E2	24.93600	14.39681	156.2122	280.1011	198.23	246.12
3	3	58.9433	7.02721	4.05716	41.4868	76.3999	52.30	66.30

Descriptives

VAR00002

	N	Mean	Std. Deviation	Std. Error	95% Confidence Interval for Mean		Minimum	Maximum
					Lower Bound	Upper Bound		
4	3	1.8683E2	32.27906	18.63632	106.6423	267.0136	155.24	219.76
Total	12	1.3289E2	75.79773	21.88092	84.7291	181.0482	52.30	246.12

Test of Homogeneity of Variances

VAR00002

Levene Statistic	df1	df2	Sig.
2.411	3	8	.142

ANOVA

VAR00002

	Sum of Squares	df	Mean Square	F	Sig.
Between Groups	59721.359	3	19907.120	45.804	.000
Within Groups	3476.893	8	434.612		
Total	63198.252	11			

Post Hoc Tests

Multiple Comparisons

Dependent Variable:VAR00002

	(I) VAR00001	(J) VAR00001	Mean Difference (I-J)	Std. Error	Sig.	95% Confidence Interval	
						Lower Bound	Upper Bound
Tukey HSD	1	2	-150.53000*	17.02178	.000	-205.0397	-96.0203
		3	8.68333	17.02178	.954	-45.8264	63.1931
		4	-119.20129*	17.02178	.001	-173.7110	-64.6916
	2	1	150.53000*	17.02178	.000	96.0203	205.0397
		3	159.21333*	17.02178	.000	104.7036	213.7231
		4	31.32871	17.02178	.323	-23.1810	85.8384
	3	1	-8.68333	17.02178	.954	-63.1931	45.8264
		2	-159.21333*	17.02178	.000	-213.7231	-104.7036
		4	-127.88462*	17.02178	.000	-182.3944	-73.3749
	4	1	119.20129*	17.02178	.001	64.6916	173.7110
		2	-31.32871	17.02178	.323	-85.8384	23.1810

*. The mean difference is significant at the 0.05 level.

Multiple Comparisons

Dependent Variable:VAR00002

	(I) VAR00001	(J) VAR00001	Mean Difference (I-J)	Std. Error	Sig.	95% Confidence Interval	
						Lower Bound	Upper Bound
Tukey HSD	4	3	127.88462*	17.02178	.000	73.3749	182.3944
LSD	1	2	-150.53000*	17.02178	.000	-189.7823	-111.2777
		3	8.68333	17.02178	.624	-30.5690	47.9356
		4	-119.20129*	17.02178	.000	-158.4536	-79.9490
	2	1	150.53000*	17.02178	.000	111.2777	189.7823
		3	159.21333*	17.02178	.000	119.9610	198.4656
		4	31.32871	17.02178	.103	-7.9236	70.5810
	3	1	-8.68333	17.02178	.624	-47.9356	30.5690
		2	-159.21333*	17.02178	.000	-198.4656	-119.9610
		4	-127.88462*	17.02178	.000	-167.1369	-88.6323
	4	1	119.20129*	17.02178	.000	79.9490	158.4536
		2	-31.32871	17.02178	.103	-70.5810	7.9236
		3	127.88462*	17.02178	.000	88.6323	167.1369

*. The mean difference is significant at the 0.05 level.

Homogeneous Subsets

VAR00002

	VAR00001	N	Subset for alpha = 0.05	
			1	2
Tukey HSD[a]	3	3	58.9433	
	1	3	67.6267	
	4	3		186.8280
	2	3		218.1567
	Sig.		.954	.323
Duncan[a]	3	3	58.9433	
	1	3	67.6267	
	4	3		186.8280
	2	3		218.1567
	Sig.		.624	.103

Means for groups in homogeneous subsets are displayed.

a. Uses Harmonic Mean Sample Size = 3.000.
