# Supplementary material for: Comparative Transcriptome Analysis of Onion in Response to Infection by Alternaria porri (Ellis) Cifferi
Source: Front Plant Sci. 2022 Apr 11;13:857306. doi: 10.3389/fpls.2022.857306 (PMC9036366; doi:10.3389/fpls.2022.857306)
Supplement: Supplementary file 4 [file Data_Sheet_4.ZIP › LOX9.pdf]

```
ONEWAY VAR00002 BY VAR00001
  /STATISTICS DESCRIPTIVES HOMOGENEITY
  /MISSING ANALYSIS
  /POSTHOC=DUNCAN LSD ALPHA(0.05).
```

# Oneway

**Notes**

| | | |
|---|---|---|
| Output Created | | 24-Feb-2022 12:15:01 |
| Comments | | |
| Input | Active Dataset | DataSet0 |
| | Filter | <none> |
| | Weight | <none> |
| | Split File | <none> |
| | N of Rows in Working Data File | 12 |
| Missing Value Handling | Definition of Missing | User-defined missing values are treated as missing. |
| | Cases Used | Statistics for each analysis are based on cases with no missing data for any variable in the analysis. |
| Syntax | | ONEWAY VAR00002 BY VAR00001 /STATISTICS DESCRIPTIVES HOMOGENEITY /MISSING ANALYSIS /POSTHOC=DUNCAN LSD ALPHA (0.05). |
| Resources | Processor Time | 00:00:00.032 |
| | Elapsed Time | 00:00:00.025 |

[DataSet0]

**Descriptives**

VAR00002

| | | | | | 95% Confidence Interval for Mean | | | |
|---|---|---|---|---|---|---|---|---|
| | N | Mean | Std. Deviation | Std. Error | Lower Bound | Upper Bound | Minimum | Maximum |
| 1 | 3 | 1.0000 | .00000 | .00000 | 1.0000 | 1.0000 | 1.00 | 1.00 |
| 2 | 3 | 4.4667 | .40415 | .23333 | 3.4627 | 5.4706 | 4.10 | 4.90 |
| 3 | 3 | 1.0000 | .00000 | .00000 | 1.0000 | 1.0000 | 1.00 | 1.00 |

**Descriptives**

VAR00002

| | N | Mean | Std. Deviation | Std. Error | 95% Confidence Interval for Mean | | Minimum | Maximum |
|---|---|---|---|---|---|---|---|---|
| | | | | | Lower Bound | Upper Bound | | |
| 4 | 3 | 8.4000 | .45826 | .26458 | 7.2616 | 9.5384 | 8.00 | 8.90 |
| Total | 12 | 3.7167 | 3.19825 | .92325 | 1.6846 | 5.7487 | 1.00 | 8.90 |

**Test of Homogeneity of Variances**

VAR00002

| Levene Statistic | df1 | df2 | Sig. |
|---|---|---|---|
| 4.800 | 3 | 8 | .034 |

**ANOVA**

VAR00002

| | Sum of Squares | df | Mean Square | F | Sig. |
|---|---|---|---|---|---|
| Between Groups | 111.770 | 3 | 37.257 | 399.179 | .000 |
| Within Groups | .747 | 8 | .093 | | |
| Total | 112.517 | 11 | | | |

# Post Hoc Tests

**Multiple Comparisons**

Dependent Variable:VAR00002

| | (I) VAR00001 | (J) VAR00001 | Mean Difference (I-J) | Std. Error | Sig. | 95% Confidence Interval | |
|---|---|---|---|---|---|---|---|
| | | | | | | Lower Bound | Upper Bound |
| LSD | 1 | 2 | -3.46667* | .24944 | .000 | -4.0419 | -2.8914 |
| | | 3 | .00000 | .24944 | 1.000 | -.5752 | .5752 |
| | | 4 | -7.40000* | .24944 | .000 | -7.9752 | -6.8248 |
| | 2 | 1 | 3.46667* | .24944 | .000 | 2.8914 | 4.0419 |
| | | 3 | 3.46667* | .24944 | .000 | 2.8914 | 4.0419 |
| | | 4 | -3.93333* | .24944 | .000 | -4.5086 | -3.3581 |
| | 3 | 1 | .00000 | .24944 | 1.000 | -.5752 | .5752 |
| | | 2 | -3.46667* | .24944 | .000 | -4.0419 | -2.8914 |
| | | 4 | -7.40000* | .24944 | .000 | -7.9752 | -6.8248 |
| | 4 | 1 | 7.40000* | .24944 | .000 | 6.8248 | 7.9752 |

*. The mean difference is significant at the 0.05 level.

**Multiple Comparisons**

Dependent Variable:VAR00002

| | (I) VAR00001 | (J) VAR00001 | Mean Difference (I-J) | Std. Error | Sig. | 95% Confidence Interval | |
|---|---|---|---|---|---|---|---|
| | | | | | | Lower Bound | Upper Bound |
| LSD | 4 | 2 | 3.93333[*] | .24944 | .000 | 3.3581 | 4.5086 |
| | | 3 | 7.40000[*] | .24944 | .000 | 6.8248 | 7.9752 |

*. The mean difference is significant at the 0.05 level.

# Homogeneous Subsets

**VAR00002**

| | VAR00001 | N | Subset for alpha = 0.05 | | |
|---|---|---|---|---|---|
| | | | 1 | 2 | 3 |
| Duncan[a] | 1 | 3 | 1.0000 | | |
| | 3 | 3 | 1.0000 | | |
| | 2 | 3 | | 4.4667 | |
| | 4 | 3 | | | 8.4000 |
| | Sig. | | 1.000 | 1.000 | 1.000 |

Means for groups in homogeneous subsets are displayed.

a. Uses Harmonic Mean Sample Size = 3.000.
