# Supplementary material for: Comparative Transcriptome Analysis of Onion in Response to Infection by Alternaria porri (Ellis) Cifferi
Source: Front Plant Sci. 2022 Apr 11;13:857306. doi: 10.3389/fpls.2022.857306 (PMC9036366; doi:10.3389/fpls.2022.857306)
Supplement: Supplementary file 4 [file Data_Sheet_4.ZIP › MYB.pdf]

```
ONEWAY VAR00002 BY VAR00001
  /STATISTICS DESCRIPTIVES HOMOGENEITY
  /MISSING ANALYSIS
  /POSTHOC=DUNCAN LSD ALPHA(0.05).
```

Oneway

Notes

Output Created		24-Feb-2022 12:16:12
Comments		
Input	Active Dataset	DataSet0
	Filter	<none>
	Weight	<none>
	Split File	<none>
	N of Rows in Working Data File	12
Missing Value Handling	Definition of Missing	User-defined missing values are treated as missing.
	Cases Used	Statistics for each analysis are based on cases with no missing data for any variable in the analysis.
Syntax		ONEWAY VAR00002 BY VAR00001 /STATISTICS DESCRIPTIVES HOMOGENEITY /MISSING ANALYSIS /POSTHOC=DUNCAN LSD ALPHA (0.05).
Resources	Processor Time	00:00:00.031
	Elapsed Time	00:00:00.017

```
[DataSet0]
```

Descriptives

VAR00002

					95% Confidence Interval for Mean			
	N	Mean	Std. Deviation	Std. Error	Lower Bound	Upper Bound	Minimum	Maximum
1	3	1.0000	.00000	.00000	1.0000	1.0000	1.00	1.00
2	3	3.5667	.40415	.23333	2.5627	4.5706	3.20	4.00
3	3	1.0000	.00000	.00000	1.0000	1.0000	1.00	1.00

Descriptives

VAR00002

	N	Mean	Std. Deviation	Std. Error	95% Confidence Interval for Mean		Minimum	Maximum
					Lower Bound	Upper Bound		
4	3	7.0667	.30551	.17638	6.3078	7.8256	6.80	7.40
Total	12	3.1583	2.60749	.75272	1.5016	4.8151	1.00	7.40

Test of Homogeneity of Variances

VAR00002

Levene Statistic	df1	df2	Sig.
4.705	3	8	.035

ANOVA

VAR00002

	Sum of Squares	df	Mean Square	F	Sig.
Between Groups	74.276	3	24.759	385.848	.000
Within Groups	.513	8	.064		
Total	74.789	11			

Post Hoc Tests

Multiple Comparisons

Dependent Variable:VAR00002

	(I) VAR00001	(J) VAR00001	Mean Difference (I-J)	Std. Error	Sig.	95% Confidence Interval	
						Lower Bound	Upper Bound
LSD	1	2	-2.56667*	.20683	.000	-3.0436	-2.0897
		3	.00000	.20683	1.000	-.4769	.4769
		4	-6.06667*	.20683	.000	-6.5436	-5.5897
	2	1	2.56667*	.20683	.000	2.0897	3.0436
		3	2.56667*	.20683	.000	2.0897	3.0436
		4	-3.50000*	.20683	.000	-3.9769	-3.0231
	3	1	.00000	.20683	1.000	-.4769	.4769
		2	-2.56667*	.20683	.000	-3.0436	-2.0897
		4	-6.06667*	.20683	.000	-6.5436	-5.5897
	4	1	6.06667*	.20683	.000	5.5897	6.5436

*. The mean difference is significant at the 0.05 level.

Multiple Comparisons

Dependent Variable:VAR00002

	(I) VAR00001	(J) VAR00001	Mean Difference (I-J)	Std. Error	Sig.	95% Confidence Interval	
						Lower Bound	Upper Bound
LSD	4	2	3.50000*	.20683	.000	3.0231	3.9769
		3	6.06667*	.20683	.000	5.5897	6.5436

*. The mean difference is significant at the 0.05 level.

Homogeneous Subsets

VAR00002

	VAR00001	N	Subset for alpha = 0.05		
			1	2	3
Duncan[a]	1	3	1.0000		
	3	3	1.0000		
	2	3		3.5667	
	4	3			7.0667
	Sig.		1.000	1.000	1.000

Means for groups in homogeneous subsets are displayed.

a. Uses Harmonic Mean Sample Size = 3.000.
