# Supplementary material for: Comparative Transcriptome Analysis of Onion in Response to Infection by Alternaria porri (Ellis) Cifferi
Source: Front Plant Sci. 2022 Apr 11;13:857306. doi: 10.3389/fpls.2022.857306 (PMC9036366; doi:10.3389/fpls.2022.857306)
Supplement: Supplementary file 5 [file Data_Sheet_5.docx]

***Supplementary materials***

**Comparative transcriptome analysis of onion in response to infection by *Alternaria porri* (Ellis) Cifferi**

Kiran Khandagale^1^, Praveen Roylawar^2^, Onkar Kulkarni^3^, Pravin Khambalkar^4^, Avinash Ade^1^, Abhijeet Kulkarni^3^, Major Singh^4^, Suresh Gawande^4*^

*** Correspondence:**[sureshgawande76@gmail.com](mailto:sureshgawande76@gmail.com), [suresh.gawande@icar.gov.in](mailto:suresh.gawande@icar.gov.in)

**1 Supplementary Data**

Supplementary Excel S1. All differentially expressed genes in both genotype along with their functional annotation

Supplementary Excel S2. PRGdb analysis

Zip folder 1: Statistical analysis for qRT-PCR of selected DEGs after PB infection in onion genotypes

Zip folder 2: Statistical analysis for enzyme activities after PB infection in onion genotypes

**2** **Supplementary Figures and Tables**

**2.1 Supplementary Figures**


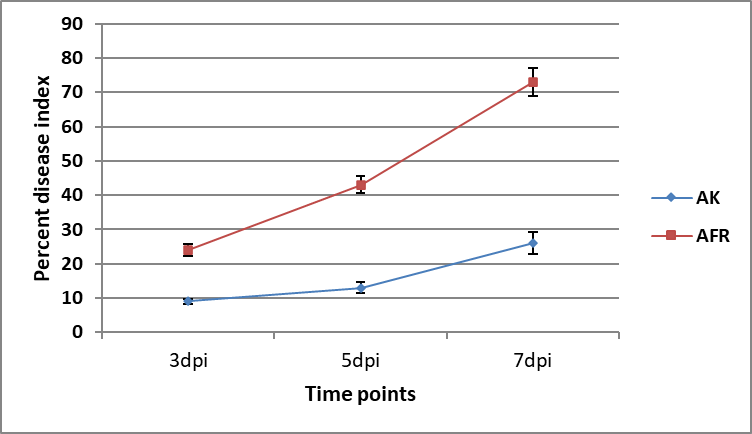


**Supplementary Figure S1.** Disease severity index of AK and AFR after *A. porri* inoculation at three time points


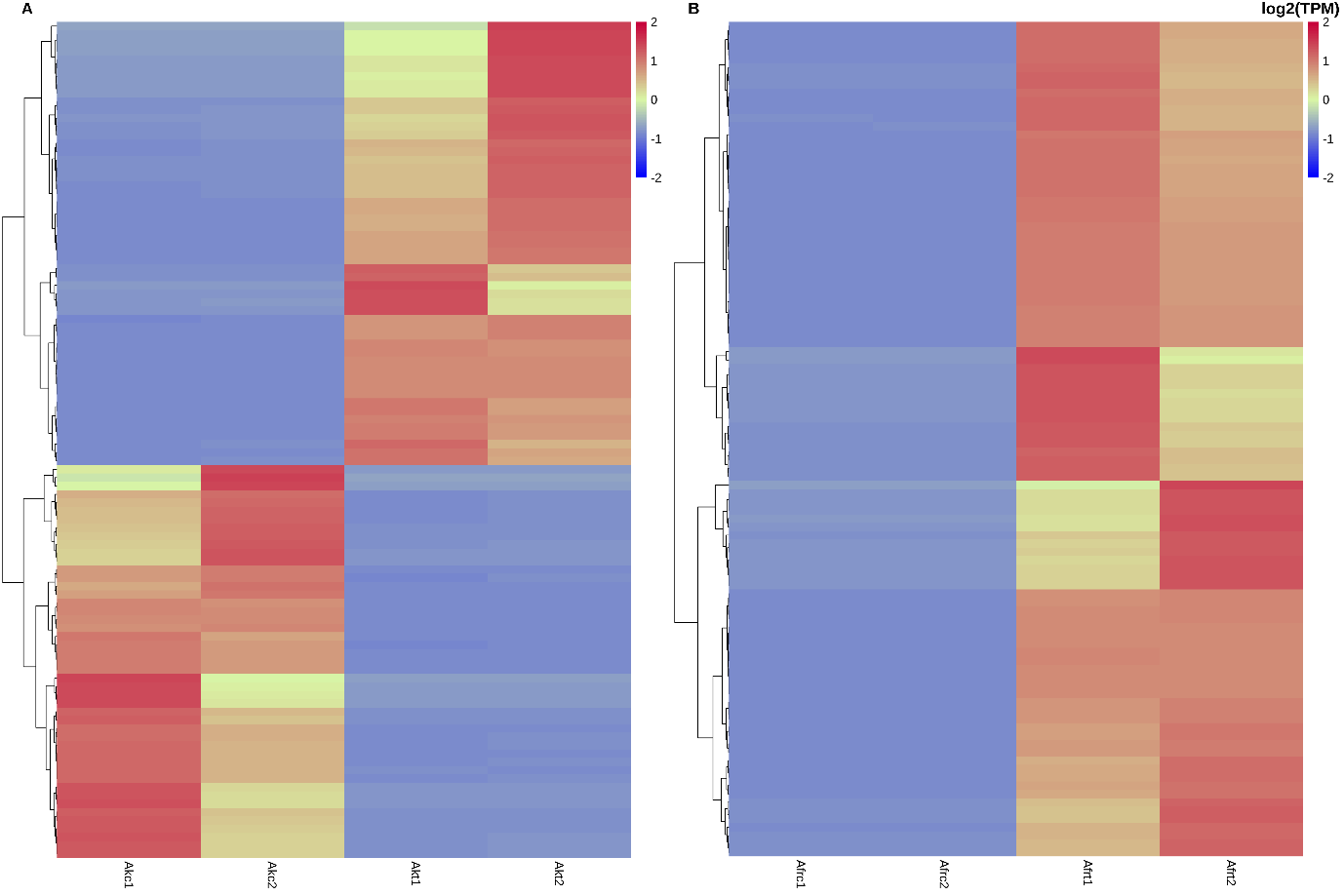


**Supplementary Figure S2.** Differential expression pattern showed by top 100 significantly expressed transcripts in onion genotypes A. Arka Kalyan and B. Agrifound rose in response to purple blotch infection


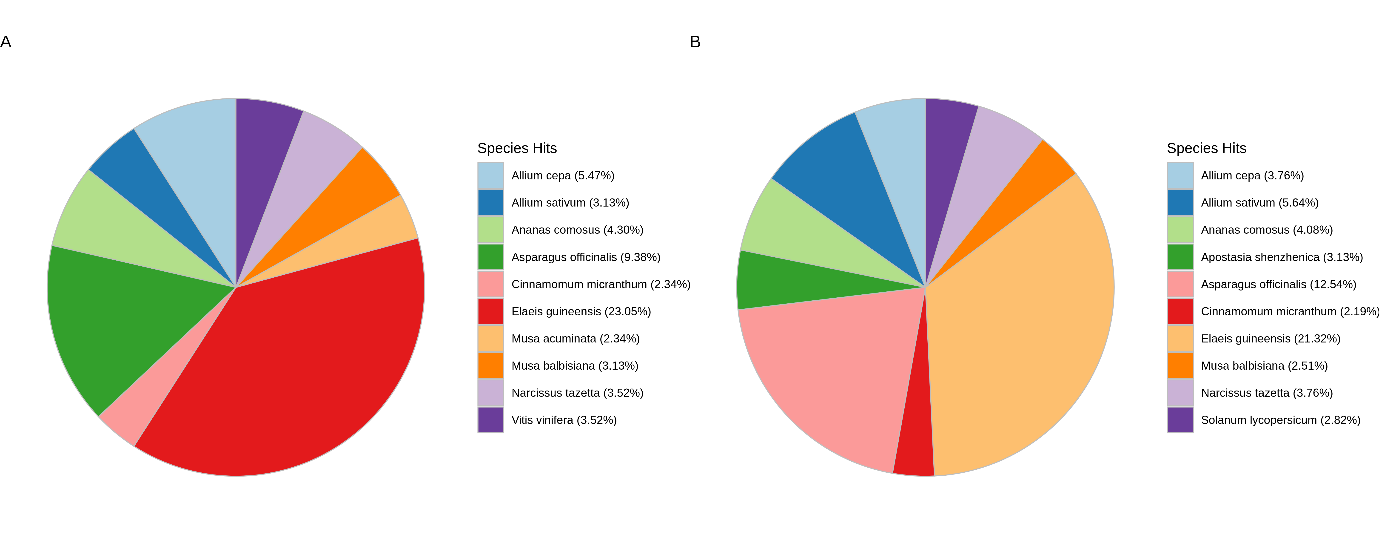


**Supplementary Figure S3.** Species hit distribution of transcripts expressed in onion genotypes; A. Arka Kalyan and B. Agrifound rose


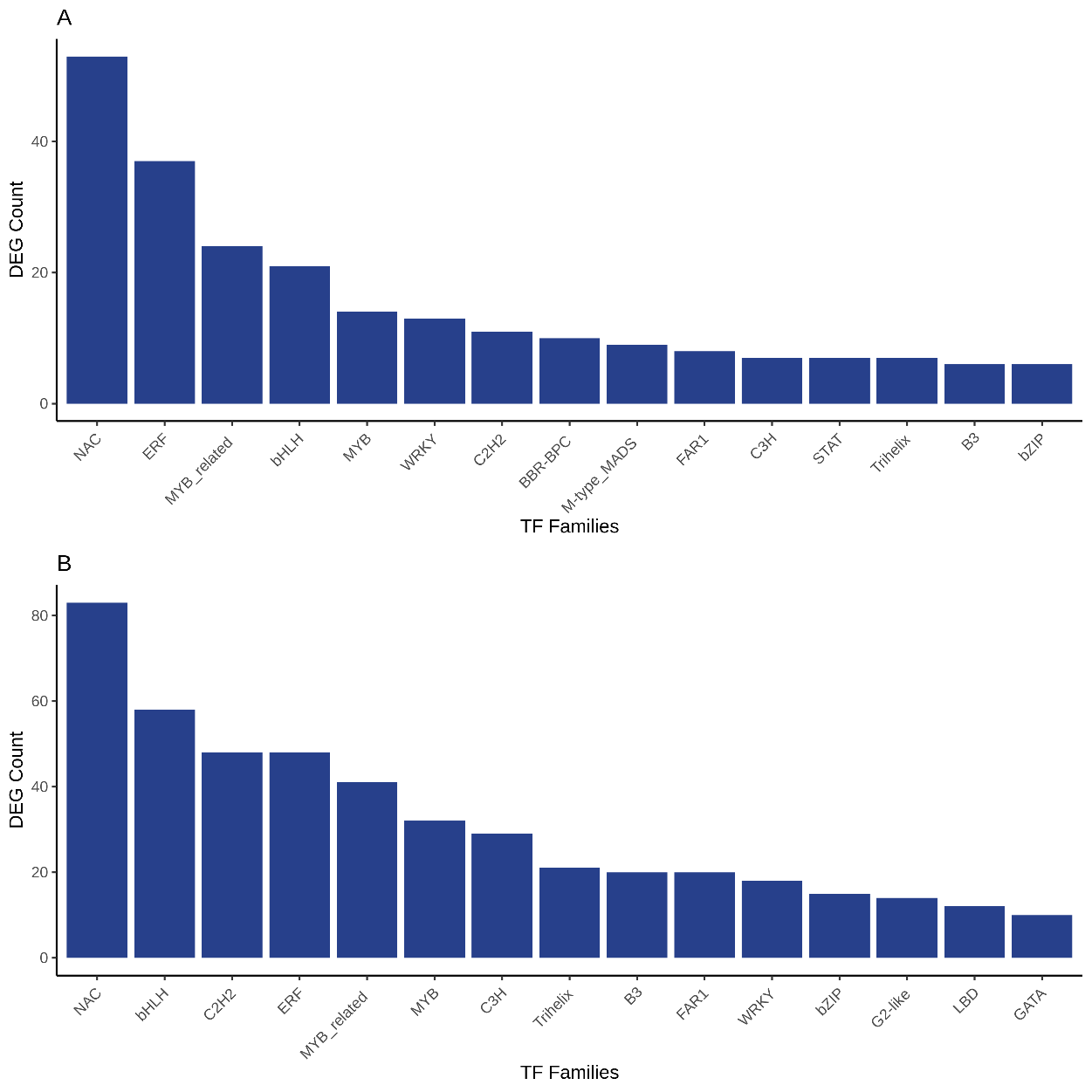


**Supplementary Figure S4.** Transcription factor distribution of transcripts expressed in onion genotypes A. Arka Kalyan and B. Agrifound rose in response to purple blotch infection. X axis represents TF families and Y axis showed DEG count for each TF family.


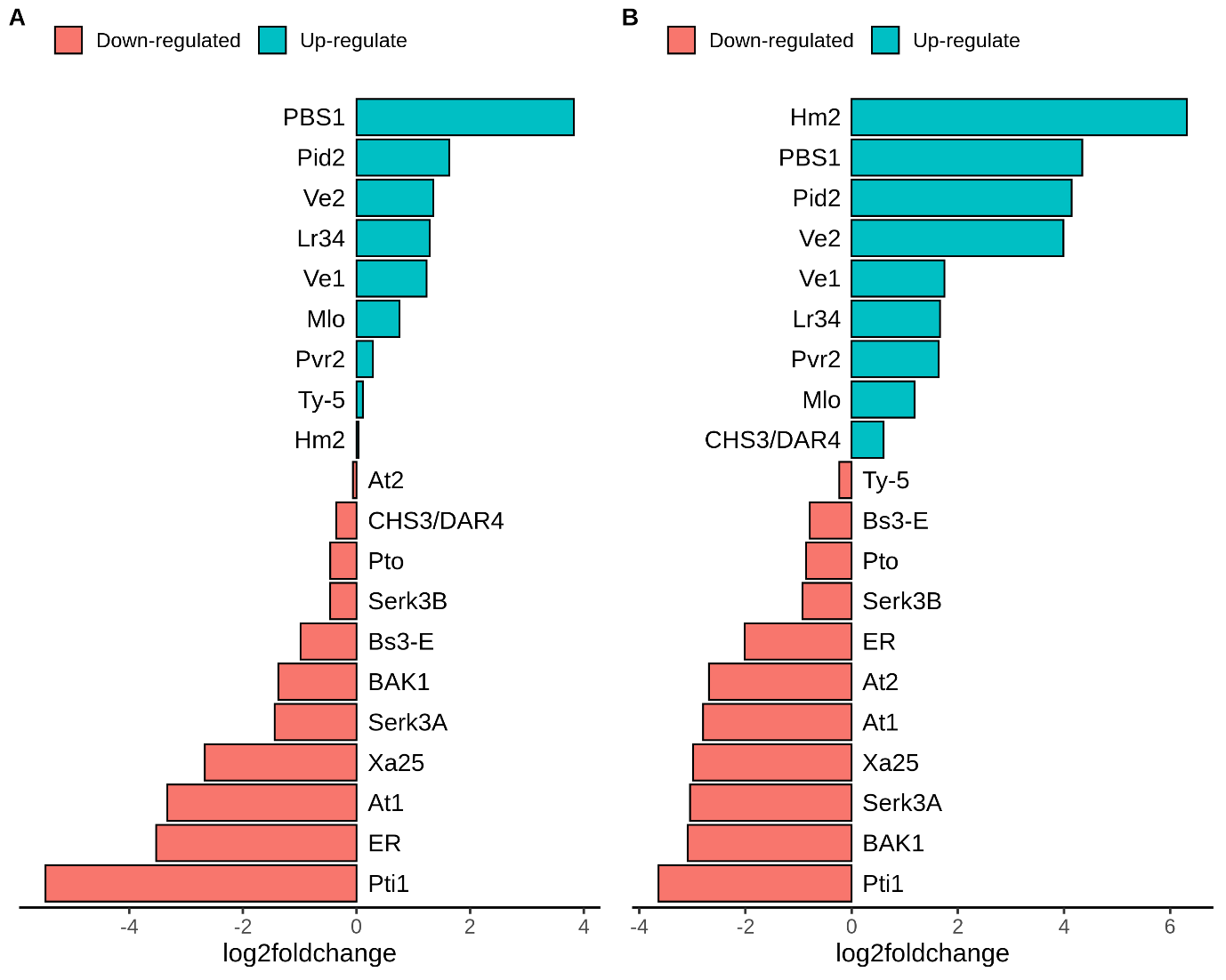


**Supplementary Figure S5.** PRGdb analysis of transcripts expressed in onion genotypes; A. Arka Kalyan and B. Agrifound rose


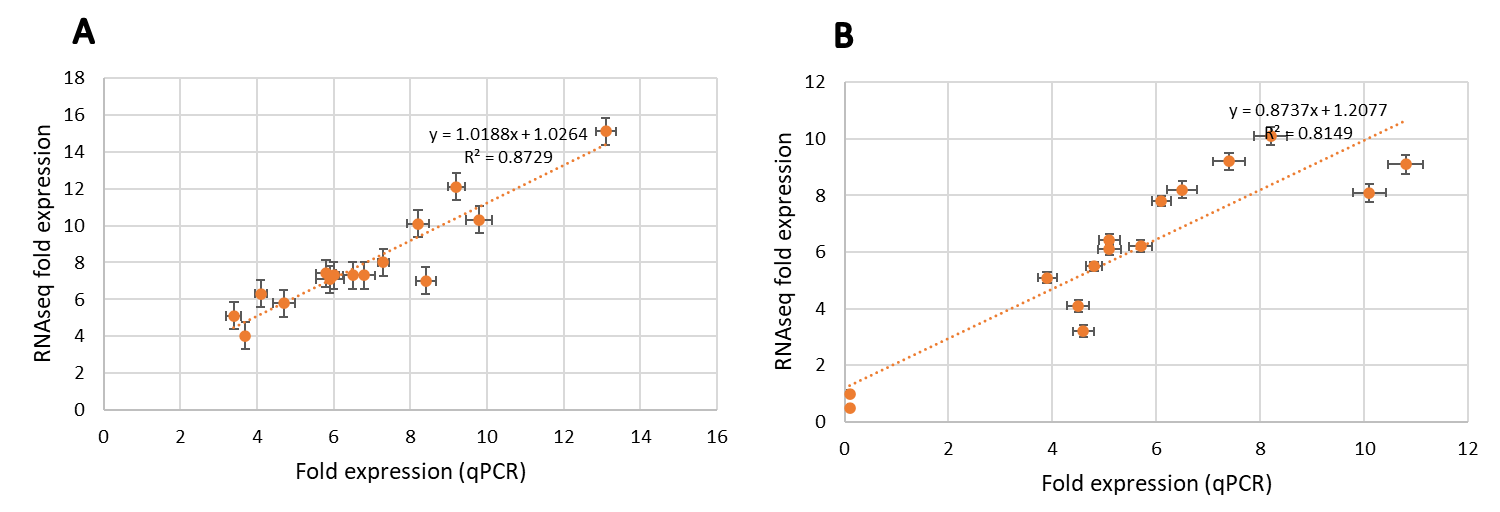


**Supplementary Figure S6.** Validation of RNA-Seq data using qPCR. A good correlation of 15 differentially expressed genes between RNA-Seq and qPCR data of Arka Kalyan (A) and Agrifound rose (B) after infection of Purple blotch

**2.1 Supplementary Tables**

**Supplementary Table 1: List of primers used for validation of RNAseq data**

| **No.** | **Name** | **Sequence (5'->3')** | **Length**  **(bp)** | **Product**  **(bp)** | **Efficiency (%)** |
| --- | --- | --- | --- | --- | --- |
| 1 | MYB_F | ACAAAGGAGTTGGGGGTTTTG | 21 | 227 | 102.4 |
|  | MYB_R | AGAGCGAACAGACAACGACA | 20 |  |  |
| 2 | ERF_F | CAAAGCGACAACAGGGGAC | 19 | 198 | 97.2 |
|  | ERF_R | GACTAGAAACGGGGTGAGGG | 20 |  |  |
| 3 | LOX_F | CTGTTCCCCATACTCGCTCA | 20 | 220 | 96.5 |
|  | LOX_R | AAGGTACTCCAGCGCAAGAA | 20 |  |  |
| 4 | PEROX_F | CAGTCCGTCCTAATCTCCCC | 20 | 247 | 104.3 |
|  | PEROX_R | ACAACCTCGGTCCACTTGAT | 20 |  |  |
| 5 | GST_F | ACAAACGCACACACGAGAAT | 20 | 230 | 103.2 |
|  | GST_R | GACGAGGTAAAGCTGCCAAA | 20 |  |  |
| 6 | PR4_F | AGTTGTTGCATCGTATGGGC | 20 | 240 | 96.7 |
|  | PR4_R | GCTCCAGTTCCTTGGTTTGT | 20 |  |  |
| 7 | PR3_F | CATGCCCTGCCAATGGATTT | 20 | 223 | 98.4 |
|  | PR3_R | TAATCGGGTGGGTTGCCTTG | 20 |  |  |
| 8 | PR5_F | GCCCATGCCTTATCCCATGT | 20 | 182 | 102.8 |
|  | PR5_R | TAATAACCCACAAGCGGCGA | 20 |  |  |
| 9 | BTB/POZ_F | TGCCGTAGACCCAAAACTTGA | 21 | 233 | 97.3 |
|  | BTB/POZ_R | GACGGAGTGGCATTAGCTCG | 20 |  |  |
| 10 | Ankyrin_F | GTCAACTACAGCACCCACCA | 20 | 209 | 99.4 |
|  | Ankyrin_R | TGGTGAAGGGAACATCTCCG | 20 |  |  |
| 11 | ACS_F | TCTCCACTCAAACGCAGCAT | 20 | 175 | 95.2 |
|  | ACS_R | ATCCAGCAAAACAAACCCGC | 20 |  |  |
| 12 | NCED_F | GCATCATCGTAGGCTGCTCT | 20 | 220 | 103.6 |
|  | NCED_R | ACGAGTTCAGGCGATTTGGA | 20 |  |  |
| 13 | NAC_F | TCCGACTCCGTTCGTAAACA | 20 | 229 | 96.3 |
|  | NAC_R | CCCGGTGTCATCACTAACCA | 20 |  |  |
| 14 | PGIP_F | TACAATCCCAGAAGCGGTCG | 20 | 184 | 98.2 |
|  | PGIP_R | AAGGGAGGTGAGGTTAGCGA | 20 |  |  |
| 15 | PR1_F | TAACCGACTTTGCCCACACA | 20 | 180 | 101.3 |
|  | PR1_F | TTCACTCCGGTGGTCCCTAT | 20 |  |  |
| 16 | *AcAct*_F | GCACCAAGAGCAGTATTC | 18 | 183 | 100.3 |
|  | AcAct_R | CCAAATCTTCTCCATGTCA | 19 |  |  |

**Supplementary Table S2: Sequencing and data QC of RNAseq of AK and AFR in response to PB**
